# A Fully In Silico Protocol to Understand Olfactory Receptor–Odorant Interactions

**DOI:** 10.1021/acsomega.4c08181

**Published:** 2025-06-03

**Authors:** Bhavika Berwal, Pinaki Saha, Ritesh Kumar

**Affiliations:** † 29742 CSIR-Central Scientific Instruments Organisation, Chandigarh 160030, India; ‡ 3769 University of Hertfordshire, Hatfield AL10 9AB, Hertfordshire, U.K.; ¶ Academy of Scientific and Innovative Research (AcSIR), Ghaziabad 201 002, India

## Abstract

Understanding olfactory receptor (OR)–odorant interaction is crucial for unraveling the molecular intricacies of smell, a sense that is essential for health and survival and has potential therapeutic applications. Nevertheless, the absence of comprehensive experimental data concerning ORs has significantly impeded the understanding of the structural dimensions of olfaction, thereby necessitating innovative approaches to elucidate the structural intricacies of ORs. In this study, we developed an in silico protocol to predict OR structures and study relevant odorant interactions using the OR51E2-propionate complex as a reference. We also developed a hybrid homology modeling strategy leveraging homologous Alphafold structures. This approach resulted in structures with better stability than Alphafold predicted models, as validated through molecular dynamics simulations. Our pipeline accurately replicated experimental findings for OR51E2 and was extended to three homologous ORs: OR51E1, OR51D1, and OR51G2. We used a total of 217 molecules from the M2OR database and key food odorants and applied K-nearest neighbor clustering, selecting a total of 78 representative molecules based on their proximity to cluster centroids for molecular docking studies. Our computational pipeline successfully verified over 25 previously established odorant–OR relationships, including the identification of potential interactions between OR51G2 and molecules such as trans-2-nonenal and acetyl glutamic acid. This framework provides an efficient method for predicting and characterizing potential OR–odorant pairs, streamlining the discovery process prior to experimental confirmation and advancing our understanding of OR binding mechanisms.

## Graphical abstract

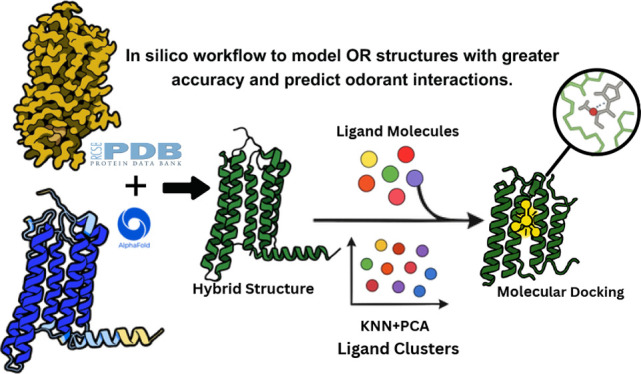

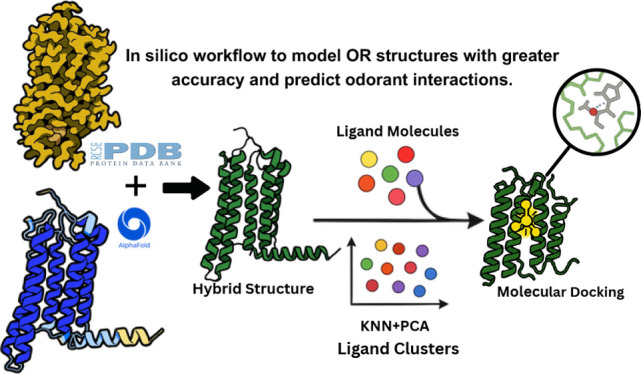

## Introduction

Olfaction is an essential biological function that profoundly impacts the behavior and survival of various species, including humans. It plays a crucial role in detecting food, identifying environmental hazards, and navigating social interactions.^1,2^ At the molecular level, olfaction is facilitated by the interaction of odorant molecules with the olfactory receptors (ORs). They are the largest family of G protein-coupled receptors (GPCRs) and are found within the Olfactory Sensory Neurons (OSNs) in the nasal epithelium.^1,3^ However, recent studies have revealed that ORs are not confined to the nasal cavity. These receptors are found in various non-olfactory tissues and organs, where they perform a myriad of biological functions that extend beyond the conventional scope of olfaction.^2,4,5^ Their presence in extra-nasal tissues suggests functions beyond odor detection, including roles in fluid balance, wound healing, digestive processes, drug metabolism, respiratory control, and potentially behavior and mood regulation.^6,7^

Olfactory perception involves about 800 ORs, out of which ∼ 400 are functional ORs, involved directly in the reception of odorants and perception of smell.^8^ Understanding olfaction at the molecular level is significant for various reasons. Knowledge of ORs and their ligands holds immense potential in practical applications. In the flavor and fragrance industries, understanding the molecular basis of scent and taste can lead to the development of enhanced products. Consequently, the study of olfaction contributes to our broader understanding of GPCRs, representing a major class of drug targets. GPCRs are also implicated in numerous physiological processes, and approximately 34% of all FDA-approved drugs target these receptors.^9,10^

The exploration of these receptors has historically been a challenge in the field of sensory biology, primarily due to their high genetic variability, limited expression in in vitro systems, and the obstacles in obtaining high-resolution structural data due to their complicated structure, making it difficult to crystallize.^11–13^ These factors have significantly impeded our ability to fully understand the structure–function relationships inherent to these receptors. Despite these challenges, there have been advances in the computational study of OR-odorant interactions that use the physicochemical properties of both the receptors and the odorants. Recent research in this domain often focuses on correlating the physicochemical properties of odorants, such as lipophilicity, molecular weight, number of double bonds, and vapor pressure, with their affinity and specificity to ORs.^3^ Mayhew et al.^14^ provided fundamental insights into how certain molecular characteristics of odorants influence their interaction with ORs revealing that lipophilicity, a measure of a compound’s affinity for lipids or fats, is a key determinant in odorant-receptor binding, affecting the compound’s ability to traverse the hydrophobic environment of the cell membrane and interact with the receptor.^15,16^

Chemoinformatic models use the known properties of odorants in the form of molecular fingerprints and their corresponding receptor responses to predict the behavior of untested odorant-receptor pairs.^17–19^ The recent development of M2OR, a comprehensive database containing over 75,000 curated OR-odorant bioassay experiments spanning 51,395 distinct pairs, has significantly enhanced our ability to validate and predict OR-odorant relationships.^20^ This extensive repository of experimental data serves as a valuable resource for validating chemoinformatic models. These methods allow the exploration of vast numbers of potential interactions that would be impractical to test experimentally. Consequently, Next-Gen Sequencing (NGS) allows for a detailed analysis of OR sequences,^21^ aiding identification of conserved domains and critical amino acids for receptor activation. These techniques have enabled us to understand ORs with higher capabilities. Given that NGS is a high-throughput technique, it typically necessitates substantial computational power, which may render it impractical for entirely disparate ORs. This often requires an exhaustive preliminary examination of chosen ORs, potentially resulting in inaccuracies.^22,23^ Molecular dynamics (MD) simulations have also provided insights on OR activation and time-resolved changes within the membrane environment, offering deeper insights into the mechanisms of receptor activation and signal transduction.^24^ Structural prediction tools like Alphafold2 (AFv2)^25^ and Alphafold3 (AFv3)^26^ have revolutionized our ability to predict the three-dimensional structures of proteins, including GPCRs, with high accuracy.

Our study capitalizes on these technological advances to investigate the structure and function of a specific subset of human ORs. Ever since the structure elucidation of the OR51E2 (PDB: 8F76),^27^ there is a possibility to model other closely related receptors with an even higher accuracy. We utilize in-silico techniques to develop a workflow, enabling us to investigate a subset of ORs, i.e., the OR51-family. Building on the experimentally determined structure of OR51E2 (PDB: 8F76), we employ a methodological approach to model other closely related receptors within this family. This is achieved by identifying receptors with high sequence similarity to OR51E2, such as OR51E1, OR51D1, and OR51G2, using BLAST searches.^28^ The high degree of sequence conservation among these receptors enhances the accuracy of homology modeling, allowing us to predict their structures reliably. Moreover, previous studies have already demonstrated the efficacy of combining Alphafold-derived structures with traditional homology modeling to enhance protein model quality. For example, an insect OR database utilized AlphaFold2-generated templates for homology modeling, significantly outperforming traditional modeling approaches such as SwissModel in terms of structural accuracy and reliability.^29^ High homology also relates to having more conserved sequences and domains, and thus a linear prediction strategy favors these receptors very well.^30,31^ Leveraging this structural information, our pipeline aims to hypothesize and identify new ligands and elucidate the functional properties of these receptors, with a special focus on OR51G2. By integrating bioinformatic analysis, structural modeling, and subsequent ligand-binding predictions, we create a comprehensive framework. We also assess the structural stability of our predicted models using MD simulations. This not only furthers our understanding of the OR51- receptor family but also opens avenues for discovering new odorant molecules and exploring their potential therapeutic and industrial applications. This approach allows for bridging the gap between the current odorant-reliant Olfactory research and the Structural Olfactory research.

## Methods

BLAST was performed on OR51E2 (PDB: 8F76) to find the OR sequences with the highest sequence similarity. Templates with sequence similarity greater than 30% are usually selected to perform homology modeling,^32^ we selected the top three most similar human olfactory receptors (hORs) to OR51E2, namely OR51E1, OR51D1, and OR51G2. The pipeline is divided into four sections, shown in Figure 1 as a flowchart.

**Figure 1:**
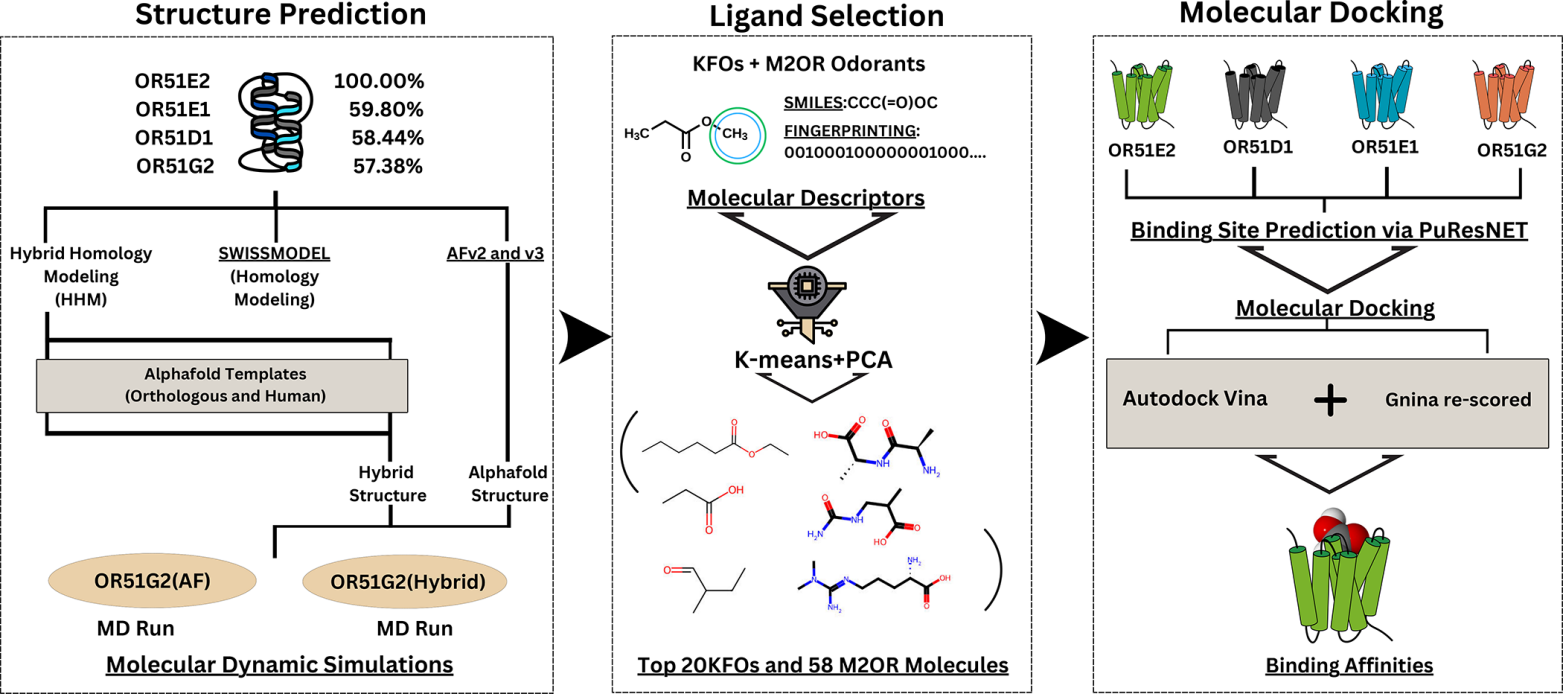
Flow-chart representing the methodology pipeline, divided into 5 phases - Structure Prediction, Binding Site Prediction, Ligand Selection, Molecular Docking, and Molecular Dynamics Simulations.

### Structure Prediction

We employ four distinct approaches to predict the structures of selected ORs. First, we obtained structures from the EBI-AlphaFold database (https://alphafold.ebi.ac.uk/) to assess AFv2-generated models under default settings. Second, we utilized AF3 to generate OR structures using default parameters.

Third, we applied traditional homology modeling via SWISS-MODEL.^33^ For OR51E1, OR51D1, and OR51G2, we used OR51E2 as the template due to its highest sequence similarity to these receptors (using pairwise sequence alignment). To model the OR51E2 itself, we employed the human Cholecystokinin A receptor (CCKAR)-Gi complex (PDB: 7EZH) as a template, despite its low sequence identity of approximately 18% with OR51E2 – well below the typical 30% threshold^30^ for homology modeling, as this was the GPCR with closest homology to OR51E2 before its structure was elucidated via cryo-EM.

Lastly, we developed a hybrid homology modeling (HHM) strategy that integrates AFv2 and AFv3 predictions with MODELER-based homology modeling.^34^ In this approach, two sets of template structures were generated: one from high-confidence mouse olfactory receptors (mORs) predicted by AFv2 and AFv3, and the other from human olfactory receptors (hORs) predicted by Alphafold (AFv2 and AFv3). Mouse OR templates were selected owing to their slightly higher pLDDT scores and high sequence homology to the target human receptors—specifically, mouse Olfr78 for OR51E2 (93% homology), Olfr558 for OR51E1 (94%), Olfr557 for OR51D1 (89%), and Olfr577 for OR51G2 (91%) [Table 1]. Mouse olfactory receptors have also been widely studied for their potential odorants, which furthers the efforts of this workflow.^8,10,35^ This selection leverages the enhanced structural quality of homologous receptors to improve the reliability of the resulting models. Additionally, AF2-predicted human OR structures were used directly as templates to serve as a benchmark. Our objective was to assess whether the use of high-confidence templates, direct human AF predictions, or their refinement via MODELER produces superior structural models. After refinement via MODELER, we further refined the hybrid structures using Rosetta Relax,^36^ a protocol designed to optimize protein structures by iteratively sampling local conformations to find the lowest-scoring variant. The protocol alternates between side-chain repacking and all-atom minimization, performing five cycles of optimization while maintaining the overall fold. This final refinement step helps to resolve any remaining steric clashes and optimize the structures according to energy functions set by Rosetta, enabling fair comparisons between models generated through different approaches. To systematically evaluate our modeling strategies, we performed a comprehensive structural analysis across 15 experimentally determined human GPCR structures [Table 2]. We calculated both unpruned and pruned RMSD values, comparing experimental structures against four different prediction methods: hybrid models built using homologous templates, hybrid models using human AF templates, and direct predictions from AFv2 and AFv3. This validation set of protein models enabled a systematic assessment of structural accuracy through RMSD calculations, providing robust insights into the relative performance of each modeling strategy. To validate, we comprehensively compared four modeling approaches—AFv2 default, AFv3 default, traditional homology modeling via SWISS-MODEL, and our hybrid method—against the experimentally determined structure of OR51E2. The evaluation was based on structural metrics such as Root Mean Square Deviation (RMSD), MolProbity scores,^37^ and Ramachandran plot analyses. MolProbity scores provide a comprehensive assessment of protein structural quality by combining clashscore, Ramachandran outliers, and unfavorable side-chain rotamers into a single metric that correlates with crystallographic resolution; lower scores indicate superior structural models. For a detailed assessment of structural quality, we employed PyRama^38^ to analyze Ramachandran plot deviations, with particular attention to Glycine and Proline residues, and used Chimera^39^ to compute RMSD values across all residues, including pruned pairs, providing a quantitative measure of model accuracy.

**Table 1:** Comparison of Human and Mouse Olfactory Receptors Showing Their Homology Percentage and pLDDT Scores from AlphaFold3 Predictions^a^

H. sapiens	M. musculus	homology	pLDDT (H. sapiens)	pLDDT (M. musculus)
OR51E1	Olfr558	94%	88.40	88.55
OR51E2	Olfr78	93%	87.40	89.72
OR51D1	Olfr557	89%	84.21	86.16
OR51G2	Olfr577	91%	87.43	88.61

a The pLDDT scores indicate the predicted confidence of the model, with higher values suggesting more reliable structural predictions.

**Table 2:** RMSD Comparisons between Different Modeling Approaches and Experimental Structures, Showing Both Unpruned and Pruned (in Parentheses) Values in Ångstroms

PDB ID	hybrid with AF templates RMSD	hybrid with human AF RMSD	AF3 vs Exp. RMSD	AF2 vs Exp. RMSD
6D9H	2.594 (0.879)	2.615 (0.871)	2.630 (0.938)	2.594 (0.879)
7AUE	1.715 (1.126)	1.760 (1.028)	2.606 (1.095)	1.760 (1.028)
7F4D	1.698 (1.121)	1.403 (0.922)	1.841 (1.034)	1.403 (0.922)
7F53	1.145 (0.949)	1.767 (0.919)	1.741 (0.777)	1.766 (0.914)
7LD3	2.374 (0.857)	2.490 (0.905)	2.554 (0.992)	2.491 (0.914)
7PIU	2.357 (0.960)	1.538 (0.929)	2.263 (0.804)	1.537 (0.903)
7WKD	1.959 (1.008)	1.961 (0.990)	1.928 (1.017)	3.359 (0.930)
7X1T	2.078 (1.061)	2.080 (1.060)	2.057 (1.021)	2.081 (1.057)
8EFB	1.539 (0.807)	1.520 (0.813)	3.173 (1.018)	1.526 (1.067)
8F7Q	1.554 (0.860)	1.588 (0.864)	3.096 (1.107)	1.560 (1.035)
8F7W	2.715 (1.105)	2.725 (1.118)	3.168 (1.109)	2.727 (0.858)
8F7X	1.835 (0.954)	3.010 (1.032)	3.205 (1.008)	3.010 (1.118)
8HDO	3.428 (1.016)	3.333 (1.006)	3.279 (0.959)	3.340 (1.033)
8HTI	1.903 (1.022)	1.905 (1.048)	2.159 (0.874)	2.008 (0.998)
8ZPS	2.454 (1.053)	2.492 (1.059)	2.565 (1.107)	2.492 (1.071)

### Binding Site Prediction

In our study, we compared and utilized multiple tools for binding site prediction to find the ligand binding pockets of the ORs and to analyze which tools perform best for these receptors. We first utilized the PUResNetv2 server, as introduced by Kandel et al.^40,41^ PUResNetv2 employs structural similarity to predict protein–ligand binding sites (PLBS) and is trained on the scPDB database, known for its annotated proteins with confirmed druggable binding sites.^42^ This tool was specifically designed for sparse proteins, i.e., proteins that have large, flexible binding pockets and undergo significant conformational changes upon ligand binding, making it a great fit for ORs and GPCRs alike. PUResNetv2 represents these proteins as Minkowski SparseTensors to efficiently capture their complex three-dimensional structures while also minimizing computational overhead, thereby preserving spatial relationships between atoms across different conformational states. It employs an encoder-decoder framework based on Minkowski Convolutional Neural Networks (MCNNs), with 171 layers and 10,861,601 trainable parameters.^41^ ORs can bind a wide variety of odorants, often in different parts of their binding pocket. PUResNetv2 is equipped with 171 layers and over 10 million trainable parameters and is well-equipped to handle this complexity and predict diverse binding sites. Finally, the focal loss function used by PUResNetv2 helps address the imbalance inherent in the protein–ligand interaction data. This is especially relevant for GPCRs, where only a small portion of the protein is directly involved in ligand binding.

We also utilized the COACH server next, introduced by Yang et al.^43^ as a complementary approach to PUResNetv2 for binding site prediction in ORs. COACH distinguishes itself as a meta-predictor, integrating PLBS results from five different methods: TM-SITE, S-SITE,^43^ COFACTOR,^44^ FINDSITE,^45^ and ConCavity.^46^ This integration allows COACH to serve as a multipurpose platform for comparing various ligand binding site predictions. It is useful for analyzing ORs and GPCRs, as it can leverage the strengths of different prediction methods to account for the large, flexible binding pockets, consisting of both orthosteric and allosteric binding sites. By combining sequence-based (S-SITE) and structure-based (TM-SITE, COFACTOR) methods with cavity detection (ConCavity) and evolutionary information (FINDSITE), COACH provides a comprehensive analysis of potential binding sites. The ability of the server to work with both experimental structures and predicted protein models makes it versatile for studying ORs, where high-resolution structures are often unavailable. This feature allows us to compare the performance of different prediction methods across various levels of structural information, providing insights into their reliability for OR binding site prediction. By using COACH alongside PUResNetv2, we aim to create a robust comparative framework, enabling us to assess the strengths and weaknesses of different approaches in the context of OR binding site prediction.

### Ligand Selection

We curated a data set comprising 62 known odorants associated with OR51E1, OR51D1, and OR51E2.^47–50^ The primary objective of this step was to verify if the hybrid models can validate experimental results and if there is a possibility of identifying new potential ligands for any of these receptors with high confidence. We note that OR51E2 is a homologue of OR51G2, showing the highest similarity to OR51E2 among all human ORs (hORs). Furthermore, Olfr577, with a 91% homology to OR51G2, suggests significant phylogenetic similarity, implying comparable functional roles. Homologous proteins within the same gene family, such as OR51G2 and OR51E2, often preserve structural and functional traits owing to their common evolutionary background. This evolutionary link may extend to the binding sites of these proteins, enabling them to engage with analogous ligands, including agonists.^51–53^

The data set with 62 known odorants was combined with another data set of 227 key food odorants (KFOs)^54^ to focus on odors that are associated with food. We extracted aliphatic organic molecules from both these data sets, resulting in a total of 151 aliphatic molecules (36 known odorants and 115 KFOs). A comprehensive molecular data set was constructed using Simplified Molecular Input Line Entry System (SMILES) strings and Extended-Connectivity Fingerprints (ECFP), along with their physicochemical properties in the form of 1-dimensional, 2-dimensional, and 3-dimensional descriptors. These properties were extracted using the RDKit cheminformatics library (https://github.com/rdkit/rdkit) and Mordred,^55^ producing a diverse array of molecular characteristics including physicochemical, topological, and structural properties. Additionally, we filtered out descriptors with a Pearson correlation coefficient greater than 0.95 to minimize redundancy and collinearity in the data set. This step ensured that only independent and meaningful descriptors were included in the analysis.

Similarly, we used the M2OR database to create a parallel data set of potential odorants. M2OR consists of OR-Odorant data based on the responsiveness of the molecule toward the odorant, making it an appropriate resource for validating computational pipelines. We extracted 102 noncyclic/aliphatic molecules with experimentally validated responses. These molecules were processed following the same pipeline as for KFOs. Following PCA-based dimensionality reduction, the M2OR-derived molecules were grouped into 5 distinct clusters. From each cluster, we selected the top 20 molecules based on their physicochemical properties and structural diversity, yielding a set of 58 molecules for subsequent molecular docking studies. This parallel analysis of M2OR data complements the KFO-based approach, providing an additional validation data set with experimentally confirmed OR-odorant interactions.

The clustering process employed a two-step approach: dimensionality reduction followed by cluster analysis. Principal Component Analysis (PCA) was first applied to the high-dimensional feature space, transforming the data into a new coordinate system oriented along the directions of maximum variance.

The PCA-reduced data were then subjected to K-means clustering, partitioning the chemical space into distinct regions. K-means aims to minimize the within-cluster sum of squares:

$$J=\overset{k}{\underset{j=1}{\sum }}\overset{n}{\underset{i=1}{\sum }}∥x_{i}^{\left(j\right)}-c_{j}{∥}^{2}$$ 1

where *x*_*i*_^(*j*)^ is the *i*-th point in cluster *j*, and *c*_*j*_ is the centroid of cluster *j*.

For K-means clustering, the maximum likelihood estimate (*L̂*) is related to this within-cluster sum of squares and can be expressed as

$$\ln(\hat{L})=\mathrm{constant}-\frac{1}{2\sigma^{2}}\sum_{j=1}^{k}\sum_{i=1}^{n}∥x_{i}^{\left(j\right)}-c_{j}{∥}^{2}$$ 2

where σ^2^ is the variance of the assumed Gaussian distributions centered at each cluster centroid.

To determine the optimal number of clusters, we implemented a statistical approach utilizing the Akaike Information Criterion (AIC)^56^ and Bayesian Information Criterion (BIC):^57^

$$\mathrm{AIC}=2k-2\ln(\hat{L})$$ 3

$$\mathrm{BIC}=\ln(n)k-2\ln(\hat{L})$$ 4

where *k* is the number of parameters, *n* is the number of data points, and *L̂* is the maximum likelihood estimate as defined above.

The robustness of the clustering solution was further validated using multiple cluster quality indices. Silhouette scores were computed to measure intracluster cohesion and intercluster separation:

$$s\left(i\right)=\frac{b\left(i\right)-a\left(i\right)}{\max\left\{a\left(i\right),b\left(i\right)\right\}}$$ 5

where *a*(*i*) is the average distance between point *i* and all other points in its cluster, and *b*(*i*) is the average distance between *i* and all points in the nearest cluster.

The Davies-Bouldin and Calinski-Harabasz indices assessed cluster compactness and distinctness, respectively:

$$\mathrm{DB}=\frac{1}{k}\sum_{i=1}^{k}\max_{j\neq i}\left(\frac{\sigma_{i}+\sigma_{j}}{d_{ij}}\right)$$ 6

$$\mathrm{CH}=\frac{\mathrm{tr}(B_{k})}{\mathrm{tr}(W_{k})}\times \frac{n-k}{k-1}$$ 7

where σ_*i*_ is the average distance of all points in cluster *i* to its centroid, *d*_*ij*_ is the distance between the centroids of clusters *i* and *j*, tr­(*B*_*k*_) is the trace of the between-cluster dispersion matrix, and tr­(*W*_*k*_) is the trace of the within-cluster dispersion matrix.

The elbow method provided visual confirmation of the optimal cluster number,^58–60^ plotting the Within-Cluster Sum of Squares (WCSS) against the number of clusters:

$$\mathrm{WCSS}=\sum_{j=1}^{k}\sum_{i=1}^{n}∥x_{i}^{\left(j\right)}-c_{j}{∥}^{2}$$ 8

Based on these analyses, five clusters were deemed appropriate. Within each identified cluster, the 20 molecules closest to the geometric center were selected as the representative compounds. The distance of each molecule to its cluster centroid was calculated using the Euclidean distance in the PCA-reduced space:

$$d_{i}=\sqrt{\overset{m}{\underset{j=1}{\sum }}{\left(x_{ij}-c_{j}\right)}^{2}}$$ 9

where *x*_*ij*_ is the *j*-th coordinate of the *i*-th molecule in the PCA space, *c*_*j*_ is the *j*-th coordinate of the cluster centroid, and *m* is the number of retained principal components.

We selected the four molecules closest to the centroids of each of the five clusters, totaling 20 molecules representing their clusters for molecular docking. This approach facilitates the selection of diverse yet representative molecules that effectively capture each cluster’s physicochemical properties. To ensure reproducibility, the complete analytical pipeline is available in Supplementary section, enabling replication of the workflow.

### Molecular Docking

Molecular docking of the selected ligands was conducted with the hybrid models of hOR51E1, hOR51E2, hOR51D1, and hOR51G2, alongside the experimental structure of OR51E2 for benchmarking. Ligand structures, sourced from PubChem in sdf format with implicit hydrogens, were converted to mol2 format using OpenBabel v3.1.2,^61^ ensuring the presence of hydrogens for accurate docking simulations. The docking process was executed in two phases to enhance the reliability of the predicted binding poses. The initial phase utilized Autodock Vina,^62,63^ which employs an empirical scoring function and efficient optimization algorithm to generate and evaluate potential binding modes. Subsequently, the binding energies of protein–ligand complexes derived from Autodock Vina were further rescored using Gnina in the second phase. Gnina, a fork of Smina and AutoDock Vina, employs a convolutional neural network (CNN)-based scoring function to refine docking predictions by evaluating binding energies. Its architecture, particularly the 'Default Ensemble' mode, consists of five carefully selected CNN models that balance performance and computational efficiency. This ensemble approach allows Gnina to learn complex, nonlinear relationships in protein–ligand interactions that may not be captured by traditional scoring functions.^64^ The use of Gnina as a secondary scoring step offers several advantages. First, its CNN-based scoring function demonstrates superior performance in both redocking and cross-docking scenarios, with benchmark tests showing significant improvements over AutoDock Vina alone. Second, Gnina’s ability to automatically learn relevant features from 3D structural data allows for a more intricate understanding of binding interactions, beneficial for the complex and diverse binding patterns of ORs. Third, Gnina provides a CNNscore for each pose, correlating well with the actual quality of the docked pose and offering an additional layer of confidence in the predicted binding modes. By combining AutoDock Vina’s robust sampling capabilities with Gnina’s advanced scoring function, we aim to generate a diverse set of initial poses and then rank these poses, providing more reliable predictions of protein–ligand interactions for our olfactory receptors. This approach is valuable when working with homology models of ORs, where the exact binding site conformations may not be precisely known. A comprehensive comparative analysis of the scores and binding energies was then conducted to evaluate the docking efficiency for each receptor–ligand pair. This dual-method approach, leveraging both traditional empirical scoring and advanced machine learning techniques, provides a more robust evaluation of potential binding modes. We also perform statistical analyses on the binding scores obtained from both Autodock Vina and Gnina. The distribution of binding energies across receptors was assessed using the Kruskal–Wallis test,^65^ a non-parametric method suitable for comparing multiple independent groups without assuming normal distribution. We also conducted ROC (Receiver Operating Characteristic) curve analysis to evaluate the predictive power of our docking approach, using experimental responsiveness data from mouse homologues (M2OR) as ground truth. The ROC analysis^66^ provides Area Under the Curve (AUC) values, which quantify how well the docking scores discriminate between active and inactive ligands. This statistical framework enables systematic comparison of docking performance across different receptors and validation against experimental data while accounting for the inherent variability in computational docking predictions.

## Results

We compared the binding behaviors of all four receptors (hOR51E1, hOR51E2, hOR51D1, and hOR51G2) for the selected odorants. We also assessed the structural stability of the OR51G2 protein models using MD Simulations. Both AFv2-generated models and hybrid models, which used AFv2 structures as templates in homology modeling, underwent a detailed protein stability evaluation. This assessment encompassed multiple analytical methods, including Ramachandran Plot analysis and MD simulations, to ensure a thorough examination of protein stability across different modeling approaches.

### Structure Prediction

In our study, BLAST analysis revealed sequence homologies of 59.8% for OR51E1, 58.44% for OR51D1, and 57.38% for OR51G2, indicating a considerable level of evolutionary conservation and suggesting potential functional or structural similarities among these proteins. We measured RMSD using the ChimeraX MatchMaker tool and calculated the RMSD over all residue pairs and over pruned pairs. The pruning process uses an iterative approach where the pairs exceeding a 2.0 Å distance cutoff are progressively removed. In each iteration cycle, the tool removes either the 10% farthest apart pairs or 50% of pairs exceeding the cutoff (whichever is fewer), then recalculates the fit, continuing until no paired atoms are more than 2.0 Å apart.^67^ This iterative pruning approach effectively excludes sequence-aligned but conformationally dissimilar regions, such as flexible loops, allowing us to focus on the best-matching 'core’ protein regions. The resulting pruned RMSD provides a more meaningful measure of structural similarity by emphasizing well-conserved structural elements while reducing the influence of locally divergent regions.

Structural analysis across 15 experimentally determined human GPCR structures is given in Table 2. The average RMSD of hybrid models with homologous Alphafold structures showed the least average RMSD (2.089 Å), followed by the hybrid-human AF template structures (2.145 Å). Notably, some structures showed method-specific performance patterns. Structure 8EFB, for example, showed marked differences between hybrid/AF2 methods (∼1.5 Å) and AF3 predictions (3.173 Å). Similarly, structure 7WKD demonstrated comparable performance across three methods (1.928–1.961 Å) but significantly higher RMSD with AF2 (3.359 Å). These findings suggest that while all methods can generate reliable GPCR structure predictions, their performance may vary depending on specific structural features. Additionally, analysis of structural models of ORs revealed that the hybrid models, specifically AF3-hybrid model as the closest representation of the experimental structure, with a root mean squared deviation (RMSD) of 2.125 Å overall and 1.006 Å across pruned pairs, slightly outperforming the AFv2 model (RMSD: 2.499 Å) and a generic homology Modeling approach (RMSD: 3.885 Å) as observed in Figure 2. The hybrid structure using AFv2 templates also produces results comparable to those of AFv3, however an average RMSD of 2.551 Å was observed in case of AFv3. Therefore, in certain cases, where there is a lack of structural data, or in case Alphafold iterations are expensive or unable to obtain a good RMSD, hybrid approaches can be adopted to refine structures, following Rosetta Relax. A general slight refinement can be observed using the hybrid approach in both cases, AFv2 and AFv3 hybrid models. Rosetta Relax helps decrease the number of bad angles in hybrid HMs that indicate localized deviations with bond angle geometry. This could stem from the hybrid modeling process, where the combination of template structures and computational modeling might not perfectly reconcile bond angle geometries everywhere in the structure, especially in regions where the template and target sequences diverge significantly.^34,68^

**Table 3:** MolProbity Data Obtained for Models Generated from AFv2, AFv3, and the Hybrid Homology Modeling (HHM) Method that Used High-Confidence AFv2 Mouse ORs as Templates^a^

ORs	model	MolProbity score	Cβ-Dev. [goal:0]	bad bonds [goal:<0.1%]	bad angles [goal:<0.1%]	CaBLAM out- liers [goal:<1.0%]
OR51E2	Exp.	1.82	0	0/2557	0/3302	2 (0.7%)
	AFv2	1.15	0	0/2557	6/3478 (0.17%)	1 (0.3%)
	AFv3	0.58	0	0/2638	8/3589 (0.22%)	2 (0.66%)
	AF2-Hyb	0.83	0	0/2557	3/3478 (0.09%)	1 (0.3%)
	AF3-Hyb	0.70	0	0/2528	5/3441 (0.15%)	0
OR51E1	AFv2	0.96	1 (0.33%)	0/2539	0.23	0
	AFv3	0.96	1 (0.33%)	0/2539	6/3469 (0.17%)	2 (0.66%)
	AF2-Hyb	0.99	0	0/2533	3/3450 (0.09%)	1 (0.3%)
OR51D1	AFv2	1.42	1 (0.33%)	0/2580	5/3514 (0.14%)	1 (0.3%)
	AFv3	1.58	0	0/2580	9/3514 (0.26%)	3 (0.9%)
	AF2-Hyb	0.75	0	0/2554	4/3480 (0.11%)	0
OR51G2	AFv2	1.57	1 (0.33%)	0/2520	6/3423 (0.18%)	2 (0.6%)
	AFv3	1.46	0	0/2520	3/3423 (0.09%)	3 (1.9%)
	AF2-Hyb	0.70	0	2/2514 (0.08%)	3/3414 (0.09%)	1 (0.3%)

a It can be observed that for almost all four receptors, the HHM method has generated a better MolProbity score. The score encompasses Clashscore, Rotamer, and Ramachandran evaluations in a single score, which then, is then normalized to be on the same scale as X-ray resolution.

**Figure 2:**
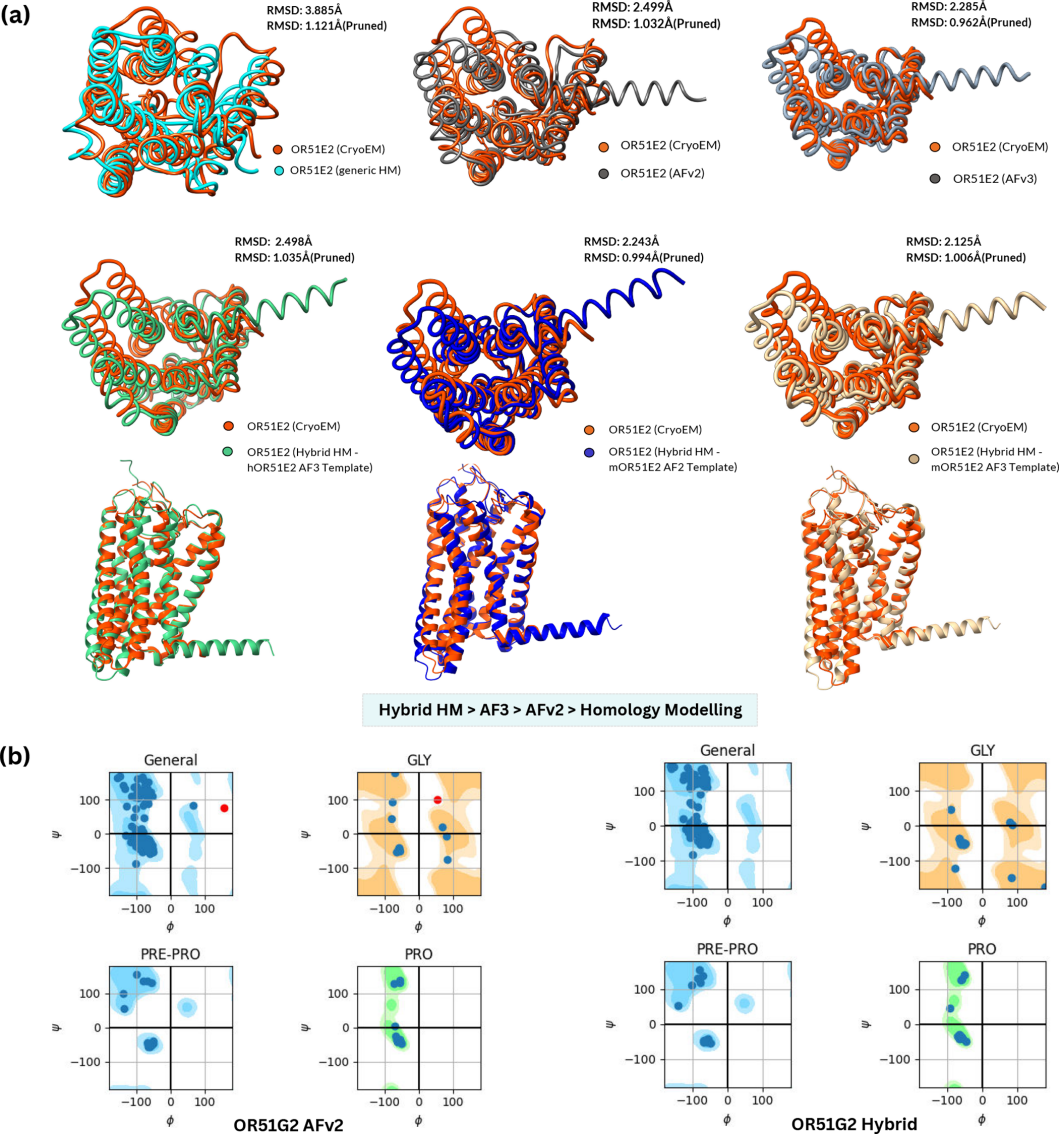
(a) RMSD values for Alpfafoldv2 and generic homology modeling performed using SwissModel and the cryo-EM structure of an activated Cholecystokinin A receptor (CCKAR)-Gi complex. On the right, showing the hybrid homology modeling structure for OR51E2, modeled using the Olfr78 structure from AFv2, hybrid structures from mouse and human ORs, and AFv3 structures with an AFv3-hybrid comparison. Pruned pairs are the subset of atom pairs retained after removing mismatched/unsuitable atoms, ensuring more accurate RMSD calculation (e.g., RMSD between 219 pruned atom pairs is 1.019 Å and across all 302 pairs: 2.238). (b) Ramachandran plots for the two protein models in focus, OR51G2. AFv2 models show outliers­(in red), while the hybrid model has its residue data within the constraints.

Analysis of MolProbity scores across the four ORs for three models, as a general rule, was compared between AFv2, AFv3, and AFv2-hybrid (see Table 3). For OR51E2, while the experimental structure showed a MolProbity score of 1.82, both hybrid approaches demonstrated superior scores (AF2-hybrid: 0.83, AF3-hybrid: 0.70), with AF3 direct prediction also showing excellent quality (0.58), while the experimental structure showed the least structural deviations. Note that the AF3-hybrid model achieves optimal stereochemistry with no CaBLAM outliers and maintains good bond angles (0.15%). For OR51G2, the AF2-hybrid model achieved a low score of 0.70, significantly better than both AF2 (1.57) and AF3 (1.46) predictions, while maintaining optimal Cβ deviations and good stereochemistry (bond angles 0.09%, CaBLAM outliers 0.3%). Similarly, for OR51D1, the AF2-hybrid approach yielded the best score (0.75) compared to direct predictions (AFv2:1.42, AFv3:1.58), with no Cβ deviations or CaBLAM outliers. The only exception was OR51E1, where direct predictions (AFv2 and AFv3, both 0.96) marginally outperformed the hybrid approach (0.99), though all models showed comparable quality metrics. These results demonstrate that hybrid approaches consistently produce models with superior stereochemistry compared to direct AF predictions, especially when coupled with Rosetta Relax for energy minimization. The slightly higher performance of hybrid methods in three out of four cases suggests that integrating AF predictions with homology modeling helps optimize local geometric quality while maintaining overall structural accuracy.

Ramachandran plot analysis was conducted to evaluate the structural quality of the protein models. Figure 2b illustrates that all residues in the hybrid models fall within the favorable conformational space, indicating a high degree of structural stability. In contrast, only two out of four of the AFv2 models demonstrated a similar distribution of residues within the favorable range. The hybrid model of OR51E1 was an exception, presenting a few outliers; however, these were notably closer to the acceptable regions compared to the outliers observed in the AFv2 models.

All our analyses point toward the greater accuracy of the hybrid models when compared with AFv2 structures in replicating the three-dimensional structures of proteins in a manner that closely mirrors their natural biological counterparts. The observed sequence homology within the OR51- family of proteins implies their phylogenetic similarity but unique biological functions, suggesting a propensity for interacting with similar receptors. This comparative study of protein structure reinforces the efficacy of hybrid modeling techniques in generating more favorable protein conformations.

### Binding Site Prediction and Ligand Selection

The predicted binding pockets were located within the orthosteric sites, making them the focal point for subsequent analyses. The predictions of PuResNET show robust behavior for OR51E2, with all potential interacting residues aligning well within the transmembrane domains TM3, TM4, TM5, TM6, and TM7 for OR51D1 and OR51G2, while TM2 was consistently not involved in binding interactions. Binding sites were also predicted via various other tools like ConCavity, FINDSITE, COACH, etc., the comparisons of the predicted binding pockets for OR51E2 are present in [Fig fig3]b, along with the results from the experimental structure. In the case of OR, our comparative analysis, shown in (Figure 3b) revealed that PUResNet consistently outperformed the integrated tools within COACH by reliably identifying orthosteric ligand binding sites for the experimental structure of OR51E2. Thus, PuResNETv2 was selected for the prediction of the binding site after comparison with COACH, as it consistently identified the orthosteric ligand binding sites in the ORs under study.

**Figure 3:**
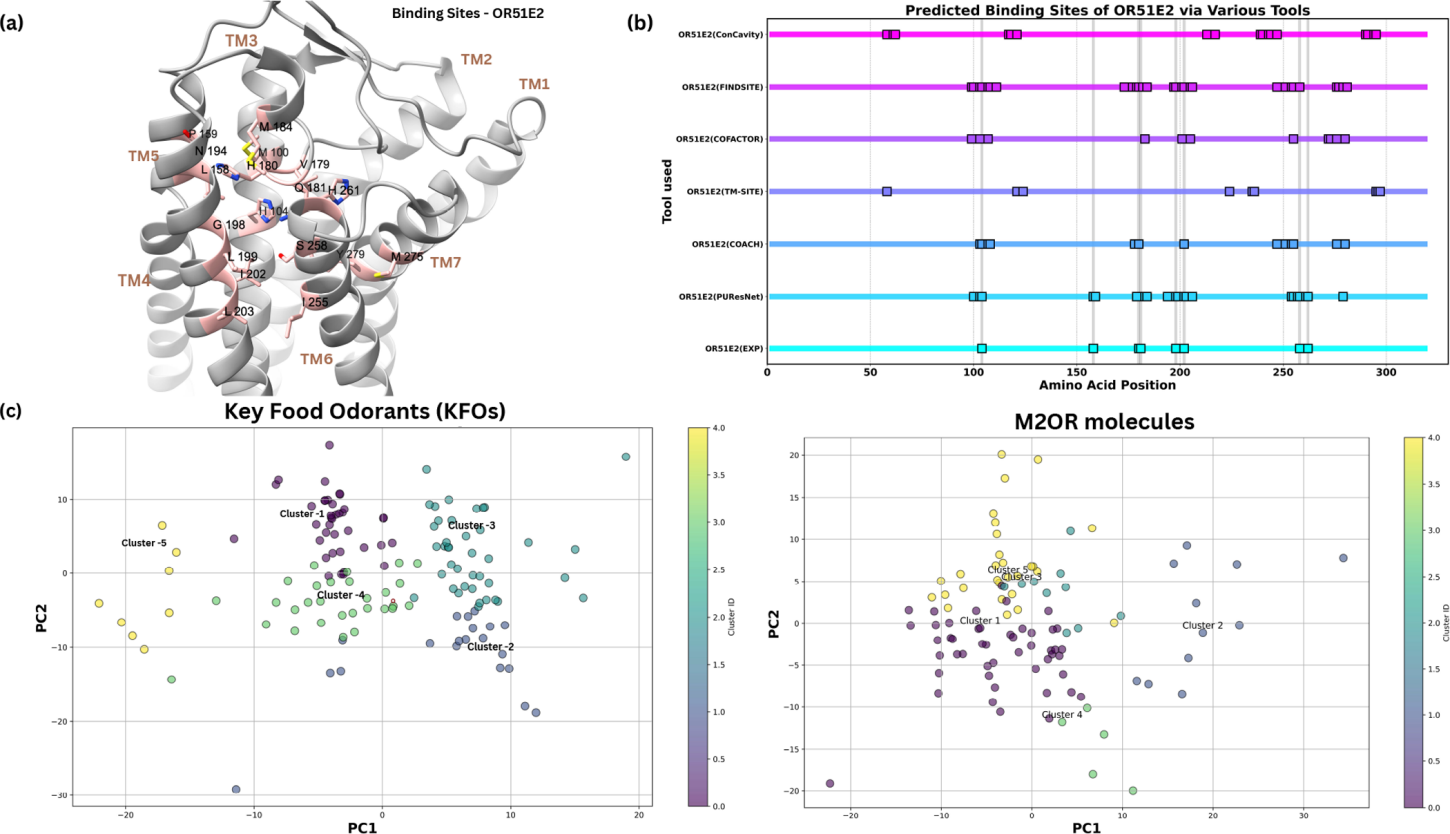
(a) Predicted binding sites in OR51E2 via PUResNet; (b) predicted binding sites of OR51E2 via various binding site prediction tools. (c) Principal component analysis (PCA) plot for the molecules shows the 5 clusters and their centroids for each data set obtained i.e. KFOs and M2OR.

The PCA of Mordred descriptors, as shown in Figure 3c, illustrates the distribution of aliphatic molecules in our data set across the chemical space. The plot reveals five distinct groups, each represented by a different color, with their centroids annotating: (Z)-non-6-enal, ethyl hexanoate, 2-methylbutanal, propanoic acid, and 1- (propyldisulfanyl) propane. The cluster predominantly contained α,β-unsaturated aldehydes with conjugated double bonds, the ethylhexanoate group comprised medium chain esters with balanced lipophilic and polar properties, the 2-methylbutanal group featured branched aldehydes, the propanoic acid group consisted of short-chain carboxylic acids and their derivatives, and the 1- (propyldisulfanyl)­propane group was characterized by sulfur-containing alkyl chains. This clustering pattern reflects the diverse chemical space of food-related odorants, ranging from highly polar carboxylic acids to more lipophilic unsaturated aldehydes. Similarly, in Figure 3c, PCA analysis of M2OR-derived molecules shows distinct clustering patterns based on their structural and physicochemical properties. The molecules are segregated into five clusters, primarily differentiated by their functional group distributions, molecular size, and polarity profiles. Clusters 1 and 5 are dominated by linear aliphatic chains containing carbonyl varying lengths, while cluster 2 features nitrogen-containing groups and mixed functionalities. Clusters 3 and 4 comprise molecules with varying degrees of unsaturation and simpler structures, respectively.

Propionic acid, visible as a centroid in the lower right quadrant of the graph, was selected as a reference compound due to its known interacting residues with OR51E2, as reported in previous studies.^27^ This selection substantiates our analysis on the basis of previously established experimental data, providing a reference point for evaluating the other ligands. From the clustering in the PCA plot, we selected 19 additional molecules to complement propionic acid. These selections were made by choosing compounds closest to each cluster centroid, ensuring a diverse representation of the chemical space. The chosen molecules exhibit a variety of olfactory characteristics and are involved in ectopic OR interactions.^3,27,69,70^ The wide distribution of points across both principal components in the PCA plot suggests the physicochemical diversity of the selected ligands. This diversity is crucial for exploring various binding modes and interactions with the olfactory receptors under study. To further characterize the selected ligands, we analyzed the correlations between their physicochemical properties (see Figure S1 in Supporting Information). The heatmap reveals strong positive correlations between molecular weight, LogP, number of rotatable bonds, and radius of gyration (correlation coefficients ranging from 0.83 to 0.98). These correlations suggest that larger molecules in our data set tend to be more lipophilic and flexible. In contrast, the quantitative estimate of drug similarity (QED) shows moderate to strong negative correlations with these properties (correlation coefficients between −0.59 and −0.74), indicating that smaller and less lipophilic molecules in our set tend to have higher drug-likeness scores. The topological polar surface area exhibits weak to moderate correlations with other properties, suggesting it captures unique structural information about the molecules. Correlation analysis of the physicochemical properties of M2OR molecules shows a strong relationship between LogP, molecular weight, and estimated boiling points (correlation coefficients >0.90), indicating that larger molecules in the data set tend to be more lipophilic with higher boiling points. The topological polar surface area showed notable negative correlations with LogP (−0.78), suggesting an inverse relationship between molecular polarity and lipophilicity (see Figure S1 in Supporting Information). These relationships align well with our understanding of odorant-receptor interactions, where both molecular size and lipophilicity play crucial roles in binding site recognition. To prepare these selected molecules for docking simulations, we performed standard preprocessing steps. Hydrogen atoms were appended to each molecule, and their protonation states were standardized to neutral. This standardization ensures consistency across all docking simulations, allowing for accurate comparisons of binding modes and energies among the diverse set of ligands.

### Molecular Docking

Docking experiments were conducted for all 78 potential ligands in the KFO data set and M2OR across all four receptor models, in addition to the experimental structure of OR51E2. Autodock Vina was used to perform molecular docking and obtain interaction energies, additionally, the energies were rescored using GNINA to observe potential docking refinements. Molecular docking was performed using GNINA and Autodock to assess ligand binding energies across four hybrid models of ORs: OR51E1, OR51E2, OR51D1, and OR51G2. The binding energies obtained from GNINA docking are visualized in Figure 4, illustrating the distinct distributions of ligand interactions among the receptors. OR51E2 exhibited the highest (least negative) binding energies, indicating weaker ligand interactions, whereas OR51D1 and OR51G2 displayed significantly lower (more negative) energy values, suggesting stronger binding interactions. OR51E1 showed an intermediate binding profile.

**Figure 4:**
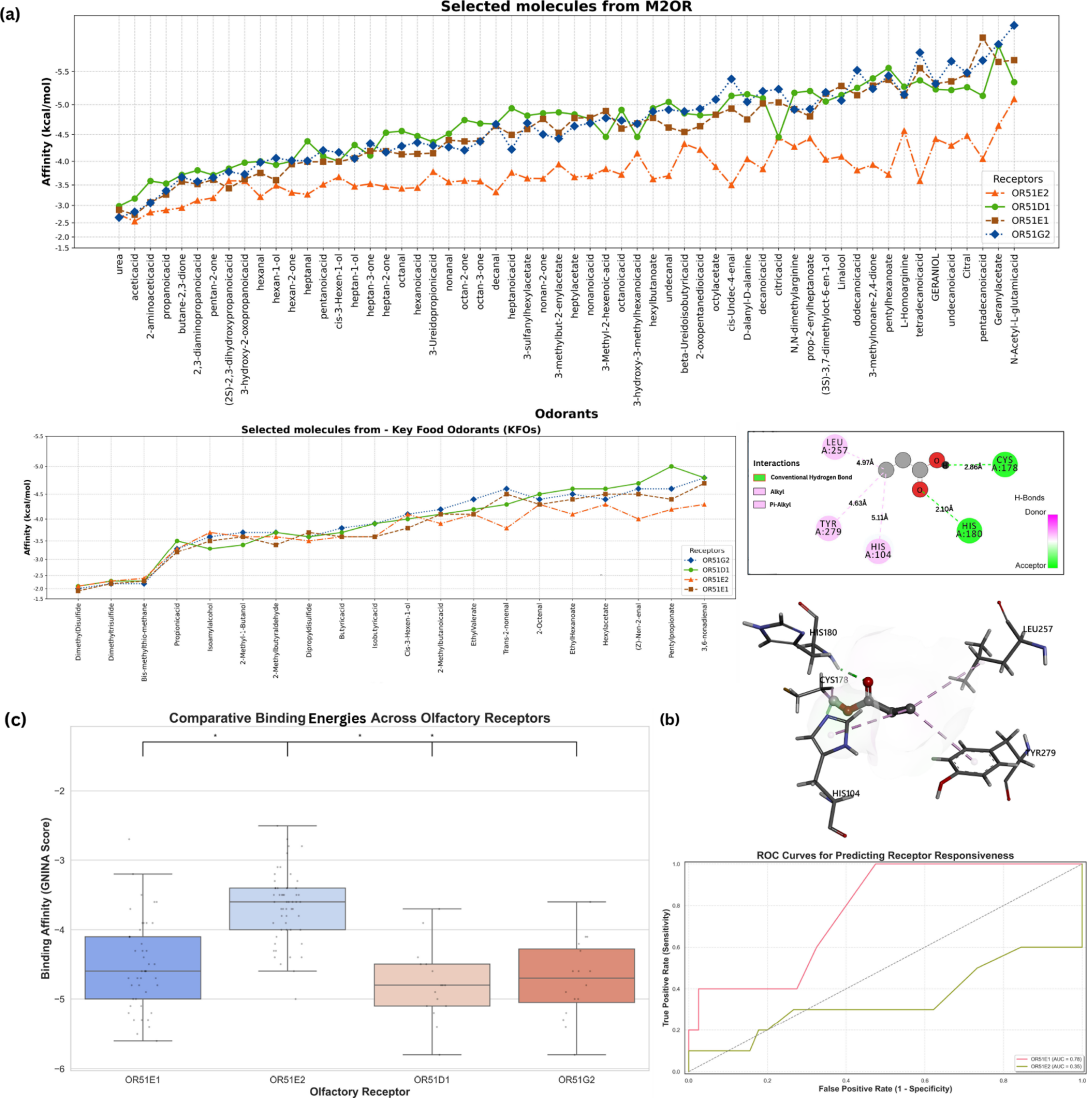
(a) Binding energies of all odorants (key food odorants and molecules from M2OR db on each hybrid receptor model. (b) Representing the predicted binding interaction of propionate with OR51E2, CYS:178, and HIS:180, forming an H-bond is apparent in the 2-D diagram on the left, and the 3-D docking pose shows the formation of these interactions. (c) The left figure compares GNINA docking energies across four olfactory receptors, where lower scores indicate stronger binding. OR51E2 shows significantly weaker energies. Statistical analysis (Kruskal–Wallis, *p* = 1.72 × 10^–12^) confirms significant receptor differences, with Dunn’s test showing OR51E2 binds significantly weaker than OR51D1 and OR51G2 (*p* < 0.0001). The Plot on the right presents the ROC curves for OR51E1 and OR51E2, the predictive performance of GNINA docking scores for receptor responsiveness. OR51E1 achieves a high AUC of 0.78, showing a strong correlation between docking scores and experimental responsiveness, whereas OR51E2 shows a significantly lower AUC of 0.35.

To statistically evaluate the differences in binding energies, a Kruskal–Wallis test was conducted, yielding highly significant results for both GNINA (*p* = 1.725 × 10^–12^) and Autodock rescoring (*p* = 1.514 × 10^–12^), confirming that binding energy distributions across receptors are not equivalent. A post hoc Dunn’s test further identified significant pairwise differences, particularly between OR51E2 and the other three receptors (refer Table 4), with OR51E2 exhibiting significantly weaker energies. To assess the reliability of binding energies as predictive metrics for ligand responsiveness, ROC curve analysis was performed for OR51E1 and OR51E2 Figure 4c. OR51E1 displayed a strong predictive correlation between docking scores and ligand responsiveness with an AUC of 0.78 (Figure 4c), whereas OR51E2 exhibited a poor predictive performance (AUC = 0.35), suggesting that docking scores alone are insufficient to predict ligand responsiveness for OR51E2. The lack of predictability for OR51E2 may be attributed to structural factors, such as alternative binding modes or ligand-induced conformational changes, that are not captured in standard docking workflows.

**Table 4:** Post Hoc Dunn’s Test Results (*p*-Values) for Binding Energy Comparisons between Receptors^a^

receptor	OR51D1	OR51E1	OR51E2	OR51G2
OR51D1	1.00	1.00	2.35 × 10^–7^	1.00
OR51E1	1.00	1.00	2.77 × 10^–8^	1.00
OR51E2	2.35 × 10^–7^	2.77 × 10^–8^	1.00	1.52 × 10^–6^
OR51G2	1.00	1.00	1.52 × 10^–6^	1.00

a Significant differences (*p* < 0.0001 are observed between OR51E2 and OR51D1/OR51G2, confirming receptor-specific binding variations.

These results indicate that virtual screening performance is receptor-dependent. While docking-based predictions are effective for OR51E1 (AUC = 0.78), they fail for OR51E2 (AUC = 0.35), suggesting that binding energy scores do not reliably predict responsiveness across all receptors. This discrepancy arises from differences in receptor binding preferences. OR51E1 and OR51D1 favor medium to long-chained fatty acids, while OR51E2 is selective for shorter to medium chains.^27,47^ Since the data set predominantly consists of medium and long-chained fatty acids, it naturally favors receptors like OR51E1, leading to better predictive performance. The poor AUC for OR51E2 suggests that docking alone is insufficient for predicting ligand interactions with this receptor. Structural differences, such as a more constrained binding pocket or specific hydrogen-bonding requirements, may contribute to this variability. These findings highlight the limitations of applying uniform virtual screening strategies across receptors.

It can be observed in Figure 4a that the hybrid OR51E2 interacted with propionate through a total of 5 residues, with 2 hydrogen bonds, i.e., between the carbonyl of propionate and HIS:180­[2.10 Å], and the hydroxyl group of propionate with CYS:178­[2.86 Å]. The presence of two hydrogen bonds suggests a strong interaction between propionate and OR51E2. The interaction energies for all molecules can be observed in Figure 4b.

In the docking results shown in Figure 5a, the OR51D1 hybrid model demonstrates strong interactions with pentyl propionate via eight residues. These interactions included two hydrogen bonds one between the alkoxy group of pentyl propionate and GLN:195­[2.13 Å], and another between the carbonyl and ARG:276­[2.34 Å], the rest of the interactions are observed to be van der Waals and pi-arene interactions, suggesting an interaction energy of −5.00 kcal/mol. Figure 5b, represents the interaction between OR51G2 and Trans-2-nonenal. The formation of two hydrogen bonds on the carbonyl group of trans-2-nonenal suggests a strong interaction with OR51G2. GLN:187­[2.01 Å] and HIS:186­[2.59 Å] both form sufficiently strong hydrogen bonds with trans-2-nonenal. Interestingly, trans-2-nonenal is associated with a strong rotten-egg, grassy smell and is a malodorous compound that is also produced naturally by the human body as a waste product of metabolism. Studies have shown that trans-2-nonenal was found to have significant negative effects on keratinocytes, as it decreases cell viability, promotes apoptosis, reduces the thickness of the epidermal layer, and decreases the number of proliferating cells.^71^ As shown in Figure 6a, OR51E1 demonstrates strong binding with citral through a combination of polar and nonpolar interactions. Two key hydrogen bonds are observed between citral’s aldehyde carbonyl and receptor residues GLN:185­[2.13 Å] and HIS:184­[2.59 Å], while hydrophobic residues (HIS:108, ILE:206, ILE:210, and PHE:257) form stabilizing pi-alkyl interactions with the ligand’s hydrocarbon chain. Similarly, geranyl acetate shows specific binding to OR51E1 (Figure 6b) through a carbon–hydrogen bond with SER:111 and multiple hydrophobic contacts involving HIS:108, PHE:257, and LEU:260, consistent with the preference of OR51E1 for medium to long-chain odorants. Notably, OR51G2 exhibits strong interactions with acetyl glutamic acid (Figure 6c), forming multiple hydrogen bonds with residues SER:264, GLN:187, HIS:186, and MET:260. The presence of these multiple short-range hydrogen bonds (2.2–2.8 Å) suggests a highly specific recognition of polar ligands by OR51G2, particularly those containing acidic and amide functionalities. Evidence exists that OR51E2 is expressed in melanocytes, and OR51B5 is also expressed in keratinocytes.^4,69,72^ Both these proteins promote wound healing and are also involved in cell migration and proliferation, apoptosis, and dendritogenesis.^4,73^ Similarly, butyric acid also shows a strong interaction with OR51E2, with an interaction energy of −3.7 kcal/mol. Butyric Acid is already a known agonist of OR51E2 as it is a short-chain fatty acid (SCFA), and OR51E2 is agonistic to these molecules.^27,35,49^ Out of our 80 tested interactions, 11 were already known through literature and experimental results (See Table S2 Supporting Information Table 2). A general observation within our data set showed that the sulfur-containing ligands dimethyl disulfide and Dimethyl Trisulfide demonstrated low binding energy across all four olfactory receptors tested (OR51G2, OR51D1, OR51E1, OR51E2).^74^ This observation does not necessarily extend to other sulfur-containing compounds or receptors outside of our selection.

**Figure 5:**
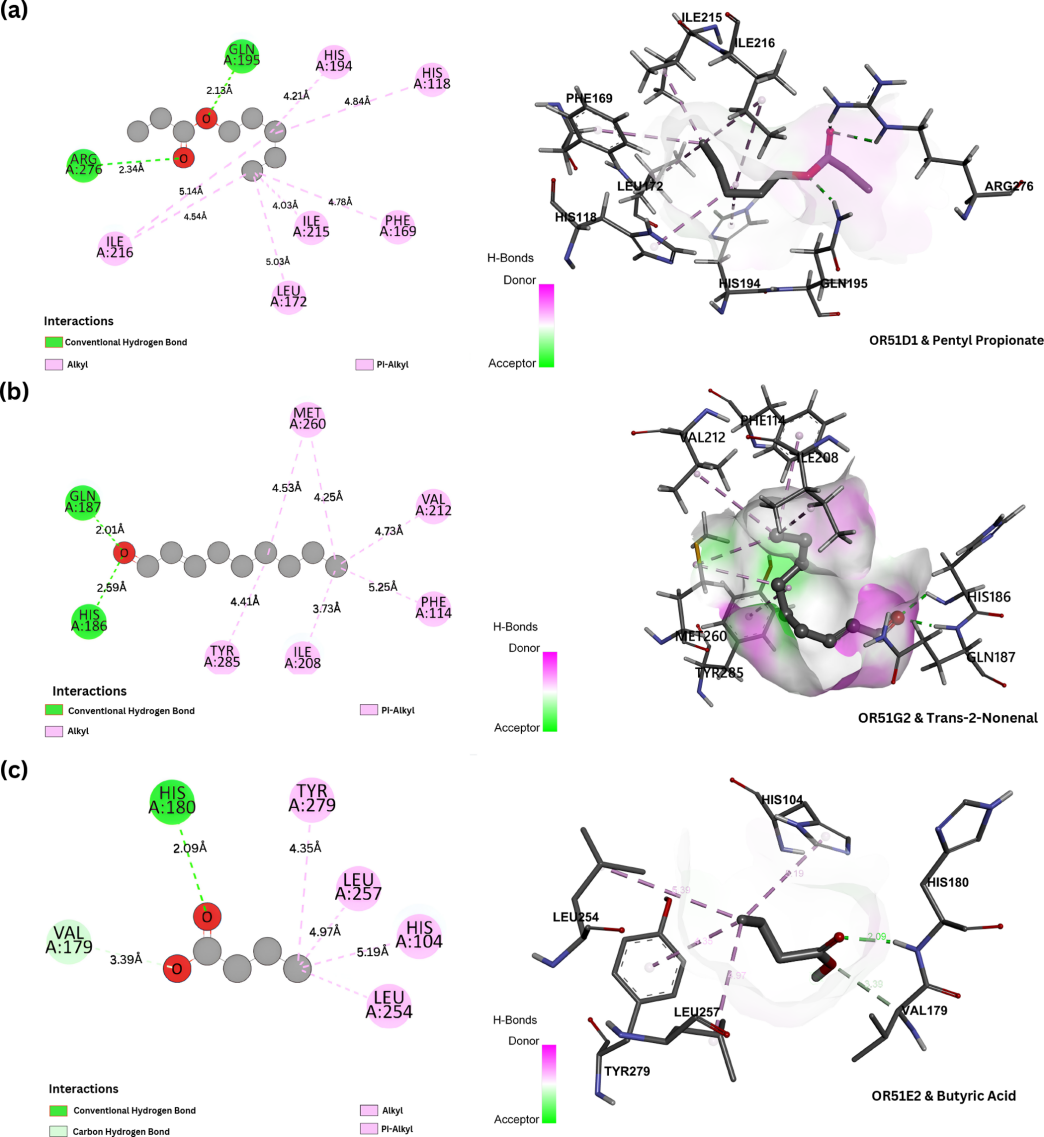
(a) Representation of the binding interaction of OR51D1 and pentyl ropionate, the highest interaction energy was obtained for this interaction and is solidified with the presence of two hydrogen-bond interactions, i.e., between the carbonyl-ARG:276, and the alkoxy-GLN:195. (b) Binding interaction between OR51G2 and 3,6-nonadienal. Regardless of the highest interaction energy with OR51G2, the OR-Odorant pair forms an unfavorable acceptor-acceptor bond; contrary to that, the same carbonyl oxygen forms a hydrogen bond with HIS:110, suggesting an avenue for stability. (c) Binding interaction between OR51E2 and butyric acid, carbonyl forms a hydrogen bond with HIS:180 and a carbon–hydrogen bond with VAL:179.

**Figure 6:**
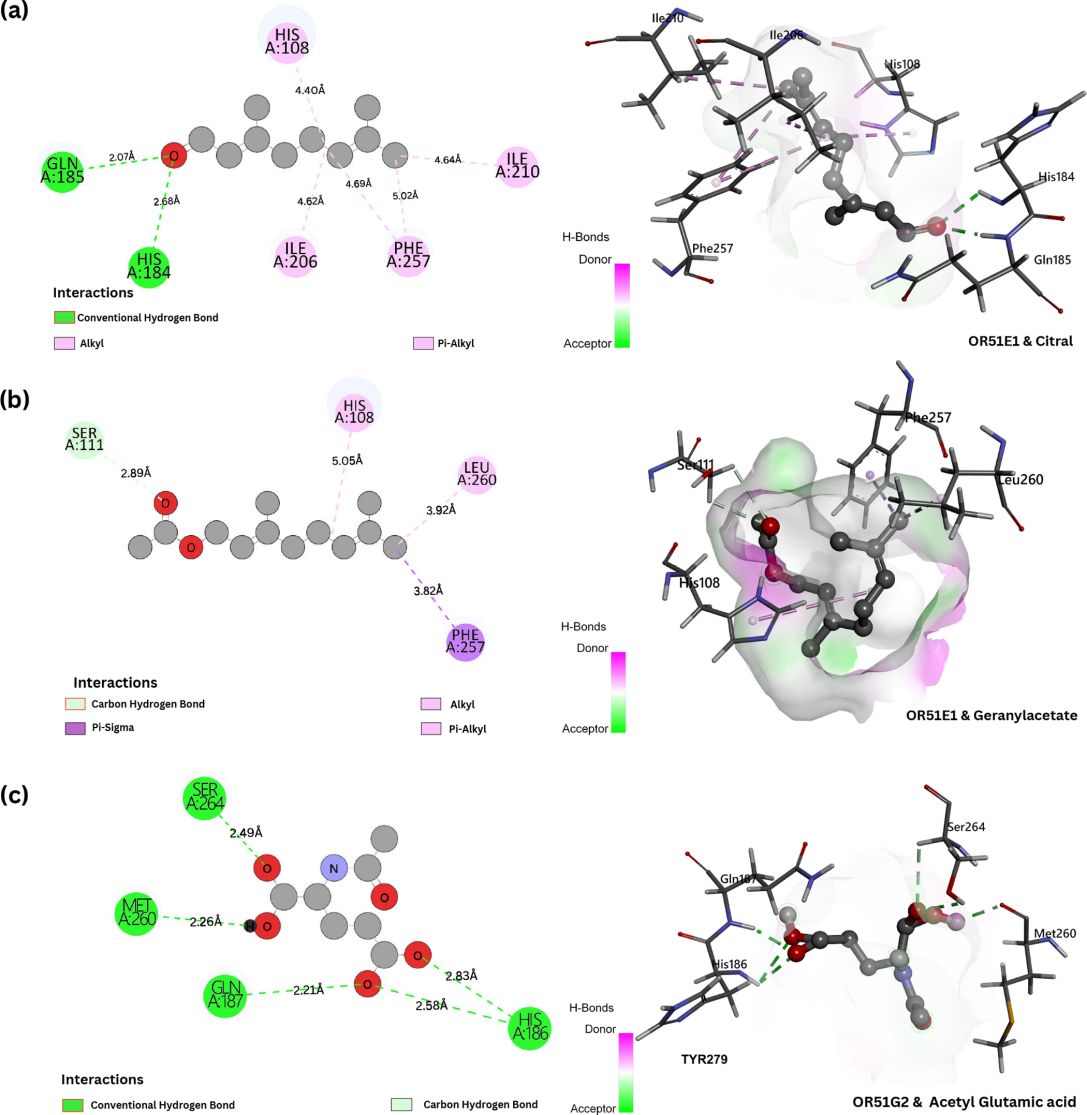
(a) Representation of the binding interaction of OR51E1 and citral, the highest interaction energy was obtained for this interaction and is solidified with the presence of two hydrogen-bond interactions, i.e., between the carbonyl-GLN:185, and the alkoxy-HIS:184. (b) Binding interaction between OR51E1 and geranylacetate. Regardless of the highest interaction energy with OR51G2, the OR-odorant pair forms an unfavorable acceptor-acceptor bond; contrary to that, the same carbonyl oxygen forms a carbon–hydrogen bond with SER:111, suggesting an avenue for stability. (c) Binding interaction between OR51G2 and acetyl glutamic acid, carbonyl forms a hydrogen bond in all its interactions and thus shows strong binding energy compared to other molecules.

### Molecular Dynamic Simulations

Molecular Dynamics (MD) Simulations were conducted on both OR51G2 models to investigate protein–membrane interaction mechanisms and determine the model with higher stability.

#### Trajectory Analysis for OR51G2

Gromacs was used to study the stability of both models after mdrun, and the RMSD across the alpha-C backbone of the GPCRs was measured using gmx_rmsd throughout runs, respectively. Three runs for each OR model were performed to optimally study the OR activity across a wider sample size. Root Mean Square Deviation (RMSD) is a critical metric used to understand the extent of deviation in molecular structures such as proteins, ligands, or their complexes from a reference structure.^75^ The calculated Root Mean Squared Deviation (RMSD) in Figure 7a (graphs represent the conformational stability of OR51G2 within a palmitoyl-oleoyl-phosphatidylcholine (POPC) lipid bilayer, as predicted by two computational models: AFv2 and a hybrid homology model. Essentially, RMSD measures how much a group of atoms has moved from its original position. Higher RMSD values indicate increased deviation from the reference structure, suggesting structural flexibility. RMSD alone does not provide insights into receptor activation or stability but reflects the extent of conformational sampling during simulations. In protein–membrane systems, fluctuations in RMSD may arise from receptor adaptation to the lipid bilayer environment rather than an inherently unstable conformation.^75,76^ To better assess the dynamic behavior of the receptor, we analyze the trajectory across multiple independent runs. The dynamic behavior of the OR51G2 receptor within the POPC lipid bilayer was assessed through three independent 100 ns molecular dynamics simulations for both the AlphaFold v2 (AFv2) and hybrid homology models. The Root Mean Square Deviation (RMSD) of the protein backbone is used as the primary metric to evaluate the conformational stability and sampling of each model.

**Figure 7:**
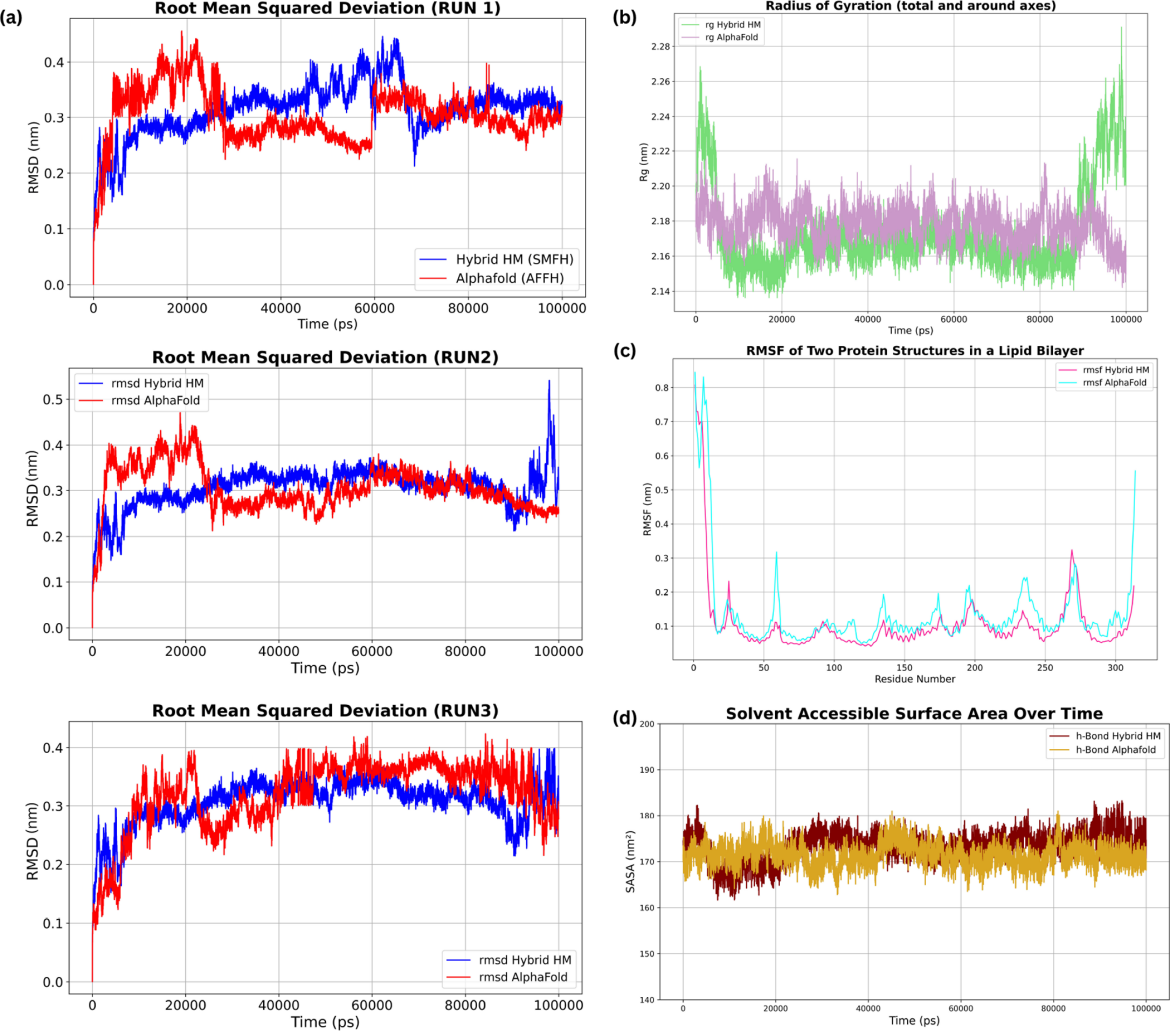
(a) Each graph shows the RMSD for three runs each for both models in comparison to each other across a length of 100 ns simulations. The system for AFv2 is observed to be more flexible initially in the first 30,000 ps compared to an overall stable hybrid model. (b) Radius of gyration across the axes of the models shows that the hybrid model tends to have more movement at the beginning and end of the simulation. (c) Root mean squared fluctuations across the receptor sequence within the lipid bilayer show increased flexibility in the ECL regions of the hybrid model, suggesting increased dynamics of the loop structures that may facilitate interactions with other proteins or the extracellular environment. (d) SASA calculations across the length of the simulation signify that the hybrid model is slightly more exposed to the solvent in the environment i.e., water.

In Run 1 (Figure 7a), the AFv2 model exhibits a rapid initial increase in RMSD, reaching a dynamic equilibrium around 0.4 nm, indicative of a significant initial relaxation or fitting of the receptor within the lipid bilayer. Some stabilization is observed around 30 ns. In contrast, the hybrid model displayed a more gradual increase in RMSD, with an increase in RMSD after 60 ns and a sudden drop around 70 ns. Beyond 80 ns, both models seem to be in a stable simulation. A different dynamic profile was evident in Run 2 (Figure 7a). Here, the AFv2 model initiated with a lower RMSD, but subsequently increased beyond the hybrid model within the first 20 ns. Such higher RMSD throughout the remainder of the simulation suggest that the AFv2 model samples a wider range of conformational states. This could be functionally important for the promiscuous binding profile of the OR. The hybrid model displayed relative stability, with a generally lower RMSD, interrupted by a singular transient spike at 80 ns, implying that the model undergoes short, significant events but remains relatively stable overall. Run 3 (Figure 7a) exhibits yet another dynamic relationship. The AFv2 model begins with a higher RMSD value than the hybrid model. The RMSD decreases over time and surpasses the hybrid model during the simulation, indicating a stable conformation during the time window. Contrary to the assumption of consistently higher RMSD values for the AFv2 model, the simulation data reveals a more nuanced picture of the dynamic behavior of both models within the lipid bilayer. While the AFv2 model displays a higher initial RMSD in Run 1, indicative of an early relaxation or fitting to the lipid environment, Run 3 exhibits a distinct trend where the AFv2 model achieves lower RMSD values for the majority of the simulation time. The hybrid model’s initial relaxation leads to an increase of RMSD, implying that AFv2 adapts to the lipid bilayer better. Notably, in Run 2, the AFv2 structure begins with a lower RMSD; however, this value rapidly increases, exceeding that of the hybrid model. These contrasting behaviors highlight the importance of considering the dynamic and stochastic nature of protein simulations.

In contrast, the hybrid homology model exhibits a more restrained, yet less consistent, RMSD profile. While the initial dynamics in Runs 1 and 2 suggest a more stable conformation relative to the AFv2 prediction, Run 3 revealed a shift in this trend, underscoring the influence of stochasticity and emphasizing the need to capture the model’s dynamic variability with a wider sampling time. The stochastic nature of the MD simulations, coupled with the dynamic interplay between the proteins and the lipid environment, introduces complexity in reaching a definitive conclusion on the dominant mode of RMSD behavior for each model. Complementary to the RMSD analysis, the radius of gyration (*R*_g_) provides insight into the overall compactness of each model (Figure 7b). The AFv2 model demonstrates relatively stable *R*_g_ values throughout the simulation, indicating resilience in its tertiary structure, potentially contributing to its functional activity. However, the hybrid homology model shows more significant variations in certain fluctuations toward the end of the simulation, specifically after 80 ns, suggesting transient unfolding events or major conformational rearrangements. Note that AFv2 has a lower *R*_g_ on average, implying that the structure is overall more compact. However, the variability in *R*_g_ indicates the presence of dynamic structural integrity, with adaptive changes within the lipid membrane, essential for receptor activation in signaling. To quantitatively describe the relative movements and deviations of the data, the average and standard deviation of both models are (hybrid: 2.19 ± 0.03, AlphaFold: 2.16 ± 0.01).

Residue-level flexibility was assessed by Root Mean Square Fluctuation (RMSF), revealing variable dynamics across both models (Figure 7c). High peaks are present in the loops of the hybrid model, suggesting increased interaction with other proteins, which might be essential for receptor functionality and downstream signaling. The comparatively lower RMSF values observed in the AFv2 model suggest a more conformationally restrained structure. Such rigidity could potentially limit the dynamic motions necessary for optimal interactions with the lipid environment, impacting processes such as membrane insertion or the formation of functional oligomeric states.^77^ The Solvent Accessible Surface Area (SASA) indicates the receptors’ hydrophobic and hydrophilic surface exposure to the lipid environment (Figure 7d). The AFv2 model exhibits overall greater SASA values, which is observed to be roughly around 170 nm^2^ during the simulation, suggesting a higher degree of exposure, with possible interactions with the solvent molecules.

To investigate whether MD simulations effectively optimized the OR structures, we compared Ramachandran plots of the AlphaFold and hybrid models before and after 100 ns MD simulations (Figure 8a). The hybrid model exhibited no Ramachandran outliers before or after MD, reflecting stable initial structural quality that was effectively preserved during simulation. In contrast, the AlphaFold model contained backbone angle outliers before MD that increased after the simulation. Figure 8 reveals two notable outliers: one in the highly unfavorable upper-right quadrant (ϕ ≈ +100°, ψ ≈ +150°) and another in the unfavorable lower-right region (ϕ ≈ +100°, ψ ≈−30°), both representing energetically disfavored conformations for typical amino acid residues. This persistent presence of outliers in the AlphaFold model likely arises from initial structural inaccuracies, such as locally trapped conformations or highly strained loops predicted by AlphaFold.^78^ Such conformations, when initially modeled incorrectly, often become trapped in local energy minima. Consequently, shorter MD simulations (e.g., 100 ns) may be insufficient in sampling the conformational landscape broadly enough to overcome the high-energy barriers and properly relax these strained regions. Typically, MD simulations can effectively relax minor structural strains, but significant initial inaccuracies or persistent local strains—often found in flexible loop regions or misfolded segments predicted by AlphaFold—require substantially longer or enhanced sampling methods to adequately resolve.^78^ This observation emphasise both the inherent robustness of our hybrid approach in generating structurally stable initial models and highlights the limitations of relying exclusively on short-duration MD simulations to resolve significant structural issues inherent in AlphaFold-generated models.

**Figure 8:**
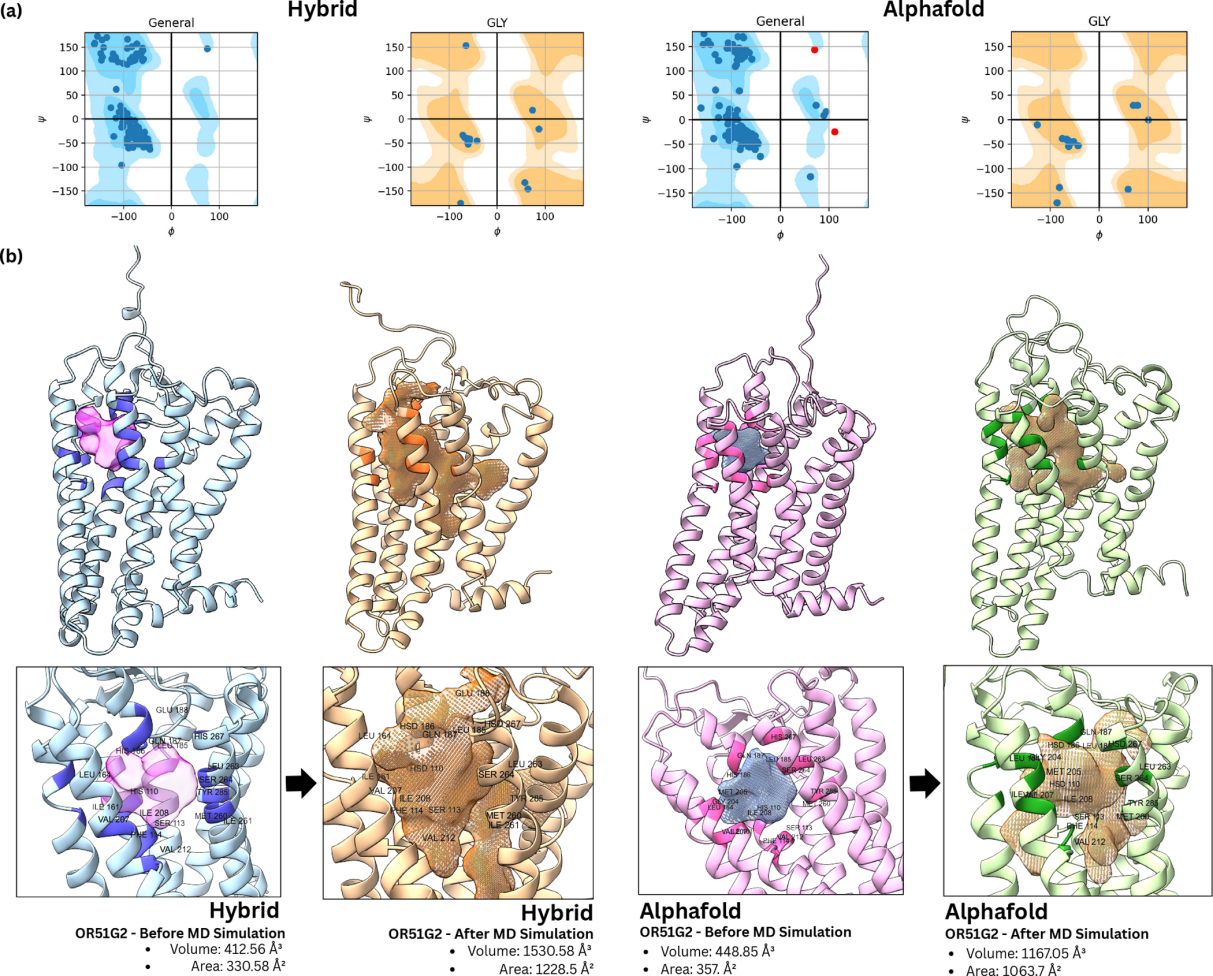
(a) Ramachandran plots for post-MD structures of the hybrid and Alphafold models. (b) Binding sites for hybrid and Alphafold models before and after MD simulations. The binding site for the hybrid model has increased relatively more than the Alphafold model by both volume and area.

The simulations also revealed significant changes in the binding site properties of the OR51G2 receptor models, particularly in terms of volume and surface area (Figure 8b). We used the KVfinder tool in ChimeraX to detect cavities or pockets in the protein structures.^79^ Both models demonstrated an expansion in binding surface area and volume, with the volume increasing by up to 270% and the surface area growing by 272%. Similarly, the AlphaFold model exhibited an increase in binding site volume by 160%, with a corresponding surface area expansion from 357 to 1063.7 Å^2^ (198%). Thus, the hybrid model shows a more pronounced expansion compared to the AlphaFold model. This difference suggests that the hybrid model possesses greater structural flexibility, likely due to enhanced conformational sampling and reorganization of residues surrounding the binding pocket during the simulation. Such flexibility may facilitate improved ligand accessibility and interaction. In contrast, the AlphaFold model exhibited a more restrained expansion, indicative of a relatively rigid structure that may limit large-scale conformational changes. While this rigidity could provide structural stability, it might also restrict adaptability for certain functional states or ligand interactions.

Overall, these results highlight the differences in structural stability and binding-site architecture between the hybrid and AlphaFold models after MD simulations. For the hybrid structure, the post-MD simulation demonstrated a significant increase in the binding pocket size (Volume: 1530.58 Å^3^, Area: 1228.5 Å^2^) while consistently maintaining favorable stereochemical properties, as evidenced by the absence of Ramachandran plot outliers both before and after MD. Although the RMSD plot showed minor variations, suggesting slight structural fluctuations when comparing pre and post MD hybrid structures, these variations did not lead to unfavorable conformations. Such minor fluctuations are expected during MD simulations because MD does not yield a single snapshot of absolute stability; instead, it captures dynamic structural transitions across a simulation period.^75,76^ Therefore, the slight RMSD variations likely indicate that the structure, while not entirely converged to a global minimum within the limited simulation time frame of 100 ns, has approached a structurally realistic and near-optimal stable conformation. Conversely, for the AlphaFold structure, post-MD analyses revealed a worsening in stereochemical quality, with an increase from one to two Ramachandran plot outliers. Despite observing an increase in the active pocket size (Volume: 1167.05 Å^3^, Area: 1063.7 Å^2^), this expansion remained comparatively smaller than that observed in the hybrid model post-MD. The persistence and even increase of unfavorable backbone angles suggest that the AlphaFold-predicted structure was structurally strained from the outset and that the 100 ns MD simulations were insufficient to alleviate these inherent structural inaccuracies. Collectively, these results suggest that the hybrid model consistently preserved high stereochemical quality while significantly improving the active pocket dimensions through MD simulations.

## Discussion

In this study, we developed a comprehensive Computer-Assisted Drug Design (CADD) pipeline to investigate olfactory receptor (OR)-odorant interactions, with a focus on the OR51 family. Our approach integrated several computational techniques, including structure prediction via various methods, molecular docking, and molecular dynamics simulations, to predict the structure and function of specific ORs and their interactions with various odorants. A key element of our structural modeling strategy was the development of a hybrid homology modeling approach, which leverages high-confidence AlphaFold (AFv2 and AFv3) predictions of both human and murine ORs as templates, followed by refinement using MODELER and Rosetta Relax. This hybrid approach resulted in structurally refined models of ORs with enhanced MolProbity scores and more favorable Ramachandran plot distributions compared to direct AlphaFold v2 or v3 predictions alone. The inclusion of Rosetta Relax, with its iterative sampling of local conformations and all-atom minimization, was crucial for optimizing the stereochemistry and resolving steric clashes in our hybrid models, leading to improved overall structural quality, especially in the loop regions connecting TM helices. To rigorously validate our modeling strategies, we performed a structural analysis across 15 experimentally determined human GPCR structures (Table 2). The hybrid models consistently yield lower MolProbity scores and more favorable Ramachandran plots, indicating improved local structural quality and stereochemistry. Furthermore, the hybrid models exhibited consistent RMSD values, a stable radius of gyration, and realistic flexibility in key regions, suggesting their suitability for further computational studies and, importantly, providing valuable templates for molecular docking studies as a downstream application. The enhanced accuracy of these models was crucial for the reliable prediction of OR-odorant interactions, as demonstrated by our successful replication of experimental findings for OR51E2.

By integrating M2OR-derived molecules into our analysis, we were able to cross-validate our computational predictions against a well-curated data set of functional responses. This approach not only enriched our ligand pool with diverse chemical structures but also strengthened our validation pipeline. The M2OR database serves as a critical resource that can help enhance the credibility of predicted OR-odorant pairs, providing a bridge between computational insights and experimentally validated outcomes. We also predicted potential ligands for the selected proteins, especially OR51G2, and found that our pipeline shows confidence in the selective selection of odorants for each receptor as OR51E2 shows a high binding energy toward shorter and aliphatic molecules like medium to short-chain fatty acids and responds relatively weakly to long-chain fatty acids.^27^ We also observe that low binding energies were obtained for sulfur molecules, further supporting the robustness and specificity of our pipeline, which is consistent with previous findings in the literature.^74^

The high binding energy of trans-2-nonenal to OR51G2 warrants further investigation into its potential functional relevance. Given the colocalization of OR51B1 expression in keratinocytes and a 55% sequence similarity between OR51B1 and OR51G2, a stronger phylogenetic relationship could be inferred, which suggests potential overlapping functionality. Trans-2-nonenal is also known to increase with aging, thus, the in silico interaction between the OR and trans-2-nonenal could be part of the mechanism by which aging affects skin health and homeostasis.^4,6,71,73^ Further experiments would clarify if such strong binding has true functional implications. Beyond this, we have also utilized various tools for binding site predictions, molecular docking, and structure prediction, performing a comparative analysis of these tools to understand which tools fit best for the study of OR-Odorant interaction. In the specific case of OR51E2, the relatively poor predictive performance observed in our docking experiments (AUC = 0.35) underscores the limitations of relying solely on binding energy scores for predicting ligand responsiveness. While OR51E1 displayed a strong correlation between docking scores and experimental responsiveness (AUC = 0.78), the discrepancy observed for OR51E2 highlights the receptor-dependent nature of virtual screening performance and suggests the involvement of more complex binding mechanisms or structural features not captured by our standard docking protocol. The differences can be better explained through the strong RMSD, the lowest molProbity scores of AF3, and the higher binding energy for AlphaFoldv2. AF3 is more compact and hence will allow a greater number of potential ligands to bind to the complex in high-throughput binding energy calculations. Post MD simulations, the structural stability of both proteins was assessed based on their final MD structures, revealing that the hybrid structure consistently maintains stereochemistry while yet an increase in the binding pocket size is observed. Whereas the AFv2 model shows an increase in binding pocket size, it fails to maintain stereochemistry, leading to comparatively lower stability. Compared to the AFv2 structure, the pocket volume of the hybrid structure grew by 270%, whereas the pocket of AFv2 grew by about 160%. This illustrates the robustness and reliability of the hybrid modeling approach in yielding biologically plausible and dynamically stable receptor models suitable for downstream computational analyses.

Several methodological limitations must be considered when interpreting our findings. First, the accuracy of our hybrid homology models relies heavily on the quality and availability of suitable homologous templates and the overall pLDDT of the Alphafold structures.^25,80^ Any deficiencies in template structures or sequence alignments can lead to inaccuracies in the predicted receptor conformations, and the benefits of Rosetta Relax refinement may be limited. Second, our docking procedures and scoring functions are, by necessity, approximations.^80,81^ As demonstrated by the low AUC for OR51E2, current docking protocols do not fully account for complex factors such as specific solvent interactions or subtle differences in receptor microenvironments, which can influence binding interactions and specificity, thus potentially leading to unreliable predictions. A major point to note is that when using AlphaFold and computational protein models for molecular docking, the binding energy predictions made by docking tools are generally lower due to the lower confidence scores of the models. Typically, a binding energy threshold around −5 kcal/mol or higher is necessary to identify potentially active compounds in virtual screening on computationally generated proteins.^80,82^ Thus, this in silico pipeline should be further validated through experimental studies with potential odorants. Third, while the M2OR database is a valuable resource for validation, it may not provide exhaustive coverage of all odorant chemotypes relevant to the receptors of interest. The odorants from KFO provide diversity to the pre-existing potential odorants obtained from M2OR. Limited chemical diversity could bias the results and restrict the generalizability of our predictions.^83^ Finally, our clustering and selection methods for identifying representative ligands depend on the chosen molecular descriptors and algorithms. These choices can skew ligand selection and potentially overlook chemotypes that fall outside established cluster centroids.

Building upon previous developments, such as iORbase that utilizes AlphaFold2-derived templates for homology modeling,^29^ our study further optimizes this approach by introducing the HHM pipeline. Our pipeline leverages high-confidence AlphaFold predictions of human and mammalian OR structures as templates, coupled with subsequent refinements through MODELER and Rosetta Relax protocols. The resulting hybrid models demonstrate significantly enhanced structural accuracy, as evidenced by improvement in overall structural stability, validated via Molprobity, Ramachandran plot distributions, and improved RMSD values compared to AlphaFold predictions alone. Furthermore, our approach addresses practical limitations associated with AlphaFold3, including constraints on the number of online-generated models and the substantial computational resources required for local installations. Consequently, our hybrid method provides a computationally accessible, resource-efficient solution, enabling quicker, large-scale, and reliable structural modeling of OR protein families. This represents a meaningful advancement over previous methods and offers a new way for common users to rapidly model a large number of OR protein structures.

In essence, the pipeline developed here offers a promising framework, but these methodological constraints underline the importance of cautious interpretation. Future improvements, including more robust template selection strategies, refined scoring functions (especially those accounting for water-mediated interactions), and more comprehensive odorant databases, will be essential for producing increasingly reliable and broadly applicable OR-odorant interaction predictions. Future studies should also focus on integrating experimental validation to confirm predicted interactions. Overall, the computational viability of our protocol is visibly high, and there is scope for further experimental studies to validate the predicted odorant-OR interactions to obtain deeper insights into these interactions. There is also potential to extend this work for in silico studies of other GPCRs and ligand interactions.

## Supplementary Material







## Data Availability

The raw data supporting the conclusions of this article will be made available by the authors without undue reservation. The code for molecular analysis, docking results, and chemical structures can be found online as a repository: https://github.com/CSIO-FPIL/OdorGenerator. Each section of the receptor and its docking results are present within each folder. The results for Ramachandran plots are also present in their designated folder. ChimeraX https://www.cgl.ucsf.edu/chimerax/ was used for molecular analysis. All the protein–membrane structures and their respective.pdb, .xtc, .gro files for each Alphafold and hybrid models related to MD Simulations can be found in https://zenodo.org/records/13376823.

## Notes

Information on the UniProt sequences used for homology modeling and structure prediction; physicochemical properties of the selected odorants; binding energy calculated for each odorant via Autodock Vina and Gnina, along with existing literature proof of known binding between the odorant–receptor pair (PDF)
S1 Data, OR-Odorant Responsiveness; S2 Data, KFOs; S3 Data, M2OR (XLSX)
OR51G2_hyb (AVI)
