# Supplementary material for: A Fully In Silico Protocol to Understand Olfactory Receptor–Odorant Interactions
Source: ACS Omega. 2025 Jun 3;10(23):24030–49. doi: 10.1021/acsomega.4c08181 (PMC12177607; doi:10.1021/acsomega.4c08181)
Supplement: Supplementary file 1 [file ao4c08181_si_001.pdf]

Supporting Information: A Fully In-Silico Protocol to Understand Olfactory Receptor-Odorant Interactions

Bhavika Berwal,[†,§] Pinaki Saha,[*,‡,§] and Ritesh Kumar[*,†,¶]

†*CSIR-Central Scientific Instruments Organisation, Chandigarh, India*

‡*University of Hertfordshire, Hatfield, Hertfordshire, United Kingdom*

¶*Academy of Scientific and Innovative Research (AcSIR), Ghaziabad, India*

§*These authors contributed equally to this work*

E-mail: p.saha3@herts.ac.uk; riteshkr@csio.res.in

Initial Structure Generation

The hybrid models were generated using MODELLER, using two templates for each receptor. For modeling the receptors OR51E1, OR51G1, and OR51D1, template 1 is OR51E2 template 2 is each respective mOR orthologs. We obtained the following structures from the following sources :

1) OR51E2 as main template for Homology Modeling: Obtained from Uniprot (Accession code: Q9H255) with sequence length of 1-320. (`https://www.uniprot.org/uniprotkb/Q9H255/entry`) **Structures for Homology Modeling:**

1. **OR51E1 (Q8TCB6):** Sequence length - 318

2. **OR51D1 (Q8NGF3):** Sequence length - 324

3. **OR51G2 (Q8NGK0):** Sequence length - 314

2) mOR AFv2 Models used for Hybrid Homology Modeling:

1. **Olfr78 (Q8VBV9):** Sequence length 320 (Q8VBV9)

2. **Olfr558 (Q8VGZ7):** Sequence length 317 (Q8VGZ7)

3. **Olfr557 (E9Q550):** Sequence length 322 (E9Q550)

4. **Olfr577 (Q8VH11):** Sequence length 312 (Q8VH11)

Selected Ligands and Docking Results

Selected ligands are shown in Table SS1 along with their major physicochemical properties. These molecules can be divided into 5 categories based on their functional groups. Molecules with higher LogP, molecular weight, topological surface area, rotatable bonds, and a higher radius of gyration show a higher binding affinity in general.

In figure S2, we evaluated cluster performance using four indices, described below: We have also made available the docking results and molecular descriptor data used in PCA as S1-Data, S2-Data and S3-Data.

- **Elbow Method (Top Left):** Shows the Within-Cluster Sum of Squares (WCSS) as a function of the number of clusters. The gradual reduction in WCSS suggests that while more clusters explain the variance better, the rate of improvement diminishes after a certain point, indicating an optimal range around 4 to 5 clusters.

- **Silhouette Score (Top Right):** Evaluates the cohesion and separation of clusters. Higher scores suggest better-defined clusters. The silhouette score peaks at 6 clusters, indicating well-separated and cohesive clusters at this point.

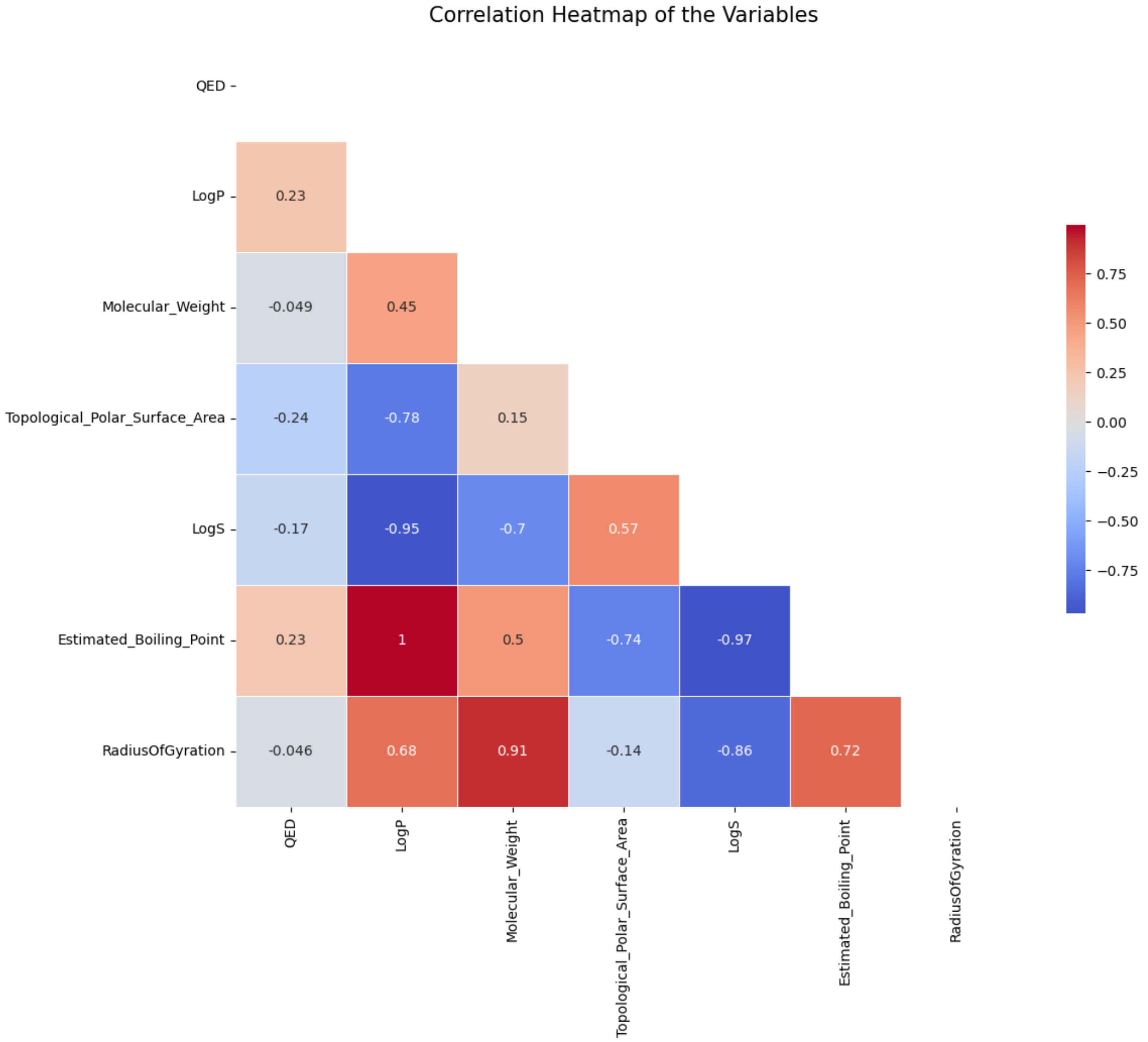

Figure S1: Heatmap to visualize the relationship between the descriptors. The correlation matrix is computed using Pearson's correlation coefficient.

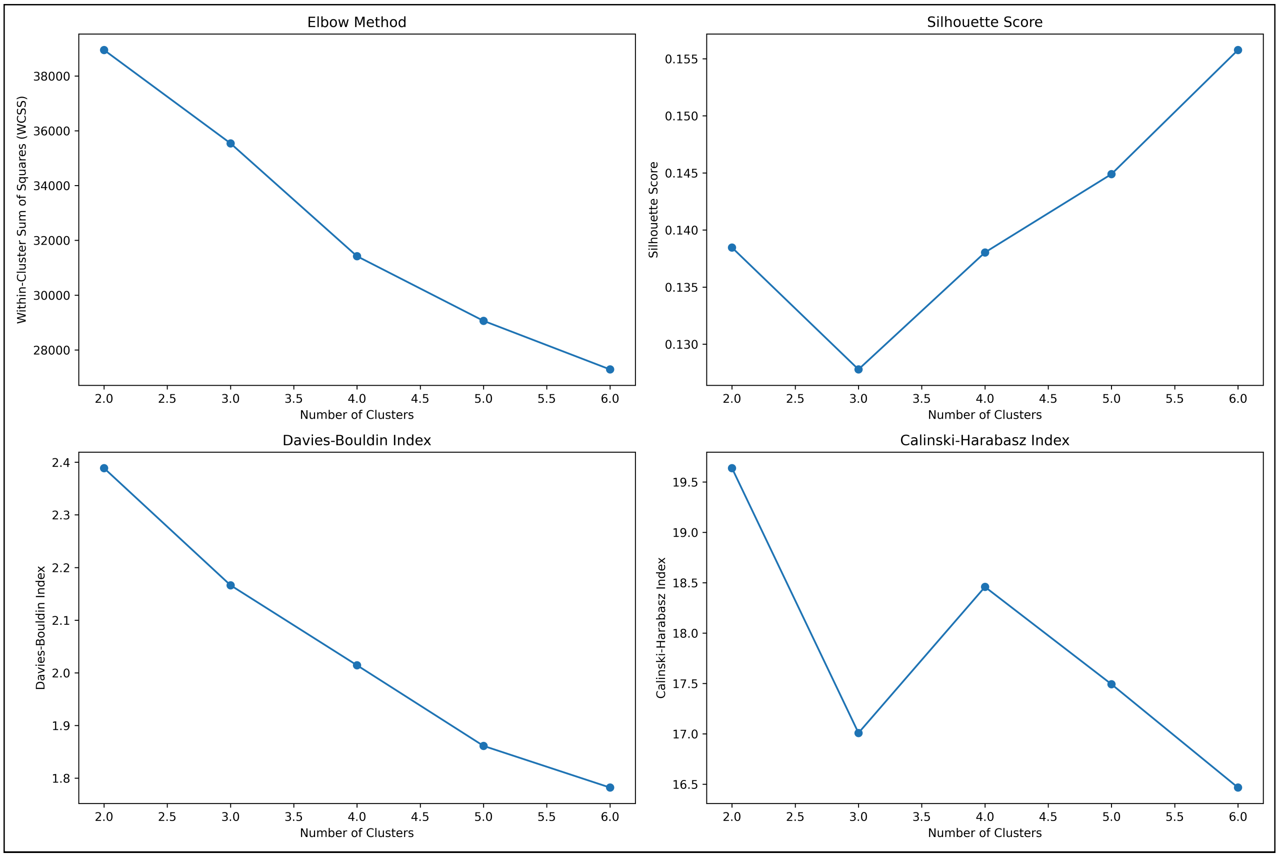

Figure S2: This figure presents the evaluation of clustering performance via various indices for different numbers of clusters (ranging from 2 to 6) using K-Means clustering on the PCA-transformed dataset.

- **Davies-Bouldin Index (Bottom Left):** Measures the average similarity ratio of each cluster with its most similar cluster. Lower values indicate better clustering. A steady decline with more clusters suggests improved clustering, with the lowest value observed at 6 clusters.

- **Calinski-Harabasz Index (Bottom Right):** Reflects the ratio of the sum of between-cluster dispersion and within-cluster dispersion. Higher values denote better-defined clusters. The highest value at 2 clusters indicates the greatest distinction between clusters, though other metrics suggest better performance with a larger number of clusters.

Together, these metrics suggest that a clustering solution in the range of 4 to 6 clusters may offer an optimal balance between compactness, separation, and overall clustering quality.

System Preparation

All structures were first pre-processed via Chimera where H-atoms were deleted. Structures for OR51G2 Hybrid and OR51G2 AFv2 were then pre-processed using CHARMM-GUI.[8,9] The receptor was positioned at the center of a rectangular prism-shaped simulation environment. This virtual box measured (100 Å) × (100 Å) × (120 Å). To mimic physiological conditions, the receptor was embedded within a palmitoyl-oleoyl-phosphatidylcholine (POPC) lipid bilayer, which served as a model cell membrane. This setup created a realistic molecular environment for studying the receptor's behavior and interactions. The receptor-embedded lipid bilayer was immersed in a water solution to mimic the natural cellular environment closely. The solution's salt concentration was set to 150 mM NaCl, replicating typical physiological conditions and standard experimental setups for G protein-coupled receptors (GPCRs). The CHARMM36m force field was employed for accurate molecular modeling to parameterize the protein, lipids, and ions. This force field is widely recognized

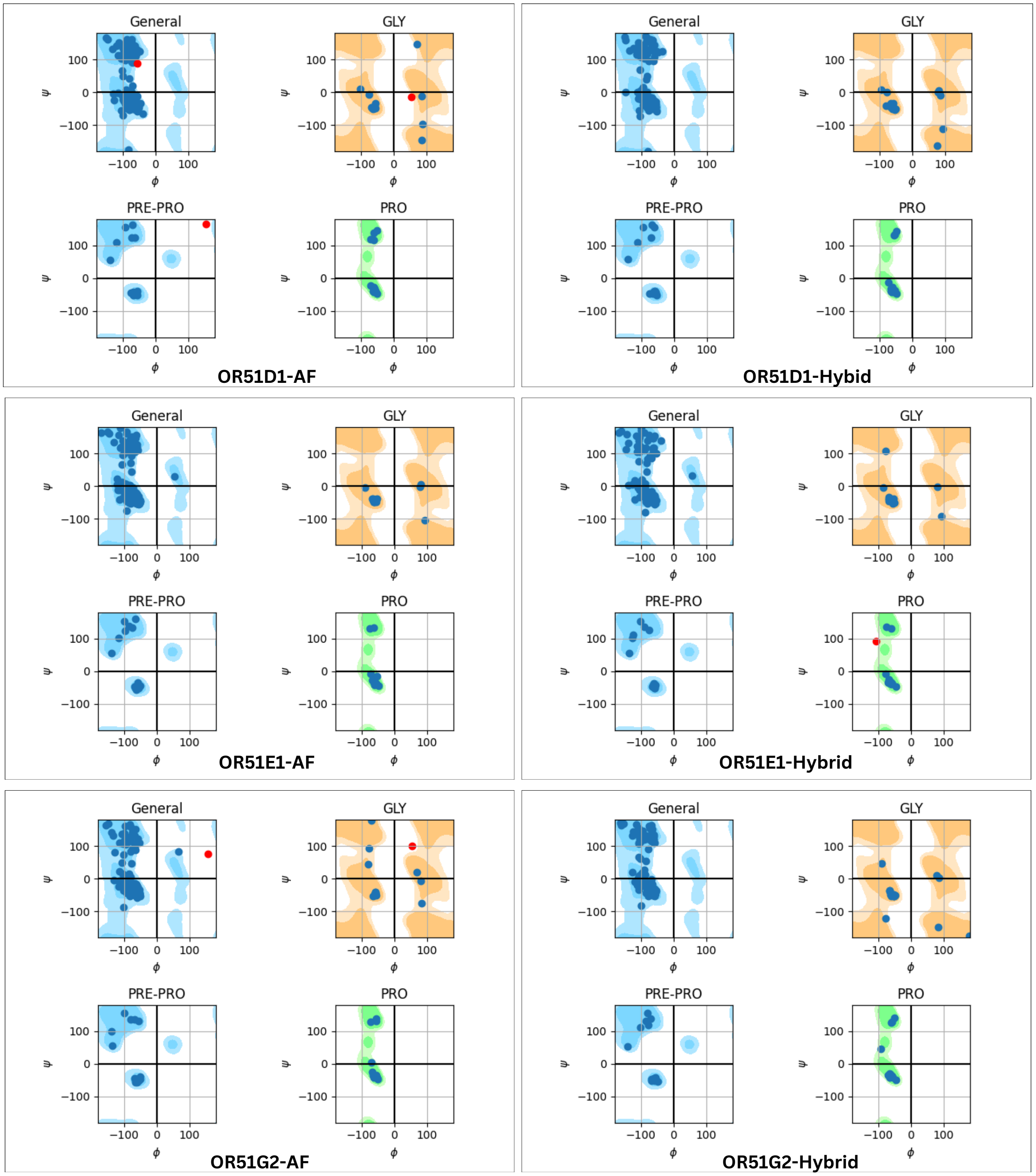

Figure S3: Plots representing the Ramachandran plots for OR51E1, OR51G2 and OR51D1. The left plots represent the plots for the AFv2 structures while the ones on right represent the hybrid structures.

Table S1: Physicochemical properties of the selected odorants - Quantitative Estimate of Drug-likeness (QED), LogP, molecular weight, topological surface area, number of rotatable bonds and radius of gyration

Common Name	QED	LogP	M.W(g/mol)	TPSA(Å^2)	Rotatable Bonds	RoG(Å)
3,6-nonadienal	0.421	2.488	138.210	17.075	3	3.187
Pentyl propionate	0.436	2.130	144.214	26.350	6	2.938
Z-Non-2-enal	0.315	2.712	140.226	17.076	3	3.158
Hexyl acetate	0.436	2.130	144.214	26.350	5	2.758
Ethyl Hexanoate	0.436	2.130	144.214	26.350	5	2.924
2-Octenal	0.314	2.322	126.199	17.075	2	2.721
trans-2-Nonenal	0.315	2.712	140.226	17.076	3	3.233
Ethyl Valerate	0.542	1.740	130.187	26.340	4	2.695
2-Methylbutanoic acid	0.567	1.117	102.133	37.320	1	1.842
cis-3-Hexen-1-ol	0.530	1.335	100.161	20.233	2	2.219
Isobutyric acid	0.513	0.728	88.106	37.310	1	1.678
Butyric acid	0.543	0.871	88.106	37.320	1	1.908
Dipropyl disulfide	0.436	3.188	150.310	0.000	5	2.703
2-Methylbutyraldehyde	0.462	1.231	86.134	17.072	1	1.719
2-Methyl-1-Butanol	0.534	1.025	88.150	20.232	1	1.827
Isoamyl alcohol	0.534	1.025	88.150	20.232	1	1.776
Propionic acid	0.491	0.481	74.079	37.310	1	1.559
Bis-methylthio-methane	0.493	1.670	108.230	0.000	2	1.986
Dimethyl trisulfide	0.521	2.276	126.270	0.000	2	2.004
Dimethyl Disulfide	0.453	1.627	94.204	0.000	1	1.601

Table S2: All docking affinities of ligands with receptors (Part 1: OR51D1 & OR51E1).

Receptor	Odorant	Affinity(kcal/mol)	GNINA re-score	Lit. Proof
OR51D1	2-Methyl-1-Butanol	-3.4	-3.52878	
OR51D1	Isoamyl alcohol	-3.3	-3.41403	
OR51D1	2-Methylbutanoic acid	-4.1	-4.22615	Yes[1]
OR51D1	Trans-2-nonenal	-4.3	-4.44884	
OR51D1	(Z)-Non-2-enal	-4.7	-4.73895	
OR51D1	2-Octenal	-4.5	-4.52	
OR51D1	Bis(methylthio)methane	-2.3	-2.33117	
OR51D1	Dimethyl Disulfide	-2.1	-2.11797	
OR51D1	Dipropyl disulfide	-3.6	-3.61061	
OR51D1	Dimethyl trisulfide	-2.3	-2.33257	
OR51D1	3,6-nonadienal	-4.8	-4.82453	
OR51D1	Ethyl Hexanoate	-4.6	-4.59272	
OR51D1	Butyric acid	-3.7	-3.7913	Yes[1]
OR51D1	Cis-3-Hexen-1-ol	-4.0	-4.06543	
OR51D1	Hexyl acetate	-4.6	-4.64702	
OR51D1	Pentyl propionate	-5.0	-4.98342	
OR51D1	2-Methylbutyraldehyde	-3.7	-3.72522	
OR51D1	Isobutyric acid	-3.9	-4.00579	Yes[1]
OR51D1	Ethyl Valerate	-4.2	-4.19817	
OR51D1	Propionic acid	-3.5	-3.54703	
OR51E1	2-Methyl-1-Butanol	-3.6	-3.64467	
OR51E1	Isoamyl alcohol	-3.5	-3.61468	
OR51E1	2-Methylbutanoic acid	-4.1	-4.17449	Yes[2]
OR51E1	Trans-2-nonenal	-4.5	-4.53383	
OR51E1	(Z)-Non-2-enal	-4.5	-4.50454	Yes[3]
OR51E1	2-Octenal	-4.3	-4.33118	
OR51E1	Bis(methylthio)methane	-2.3	-2.26416	
OR51E1	Dimethyl Disulfide	-1.9	-1.91664	
OR51E1	Dipropyl disulfide	-3.7	-3.725	
OR51E1	Dimethyl trisulfide	-2.2	-2.15917	
OR51E1	3,6-nonadienal	-4.7	-4.69426	
OR51E1	Ethyl Hexanoate	-4.4	-4.40935	
OR51E1	Butyric acid	-3.6	-3.72465	Yes[4]
OR51E1	Cis-3-Hexen-1-ol	-3.8	-3.9023	Ortholog(mouse)[3,5]
OR51E1	Hexyl acetate	-4.5	-4.48405	
OR51E1	Pentyl propionate	-4.4	-4.39643	
OR51E1	2-Methylbutyraldehyde	-3.4	-3.37326	
OR51E1	Isobutyric acid	-3.6	-3.68288	Yes[1]
OR51E1	Ethyl Valerate	-4.1	-4.08964	
OR51E1	Propionic acid	-3.2	-3.25839	

Table S3: All docking affinities of ligands with receptors (Part 2: OR51E2 & OR51G2).

**Receptor**	**Odorant**	**Affinity (kcal/mol)**	**GNINA re-score**	**Literature Proof**
OR51E2	2-Methyl-1-Butanol	-3.6	-3.72592	
OR51E2	Isoamyl alcohol	-3.7	-3.80763	
OR51E2	2-Methylbutanoic acid	-3.9	-4.00398	Yes[6]
OR51E2	Trans-2-nonenal	-3.8	-3.84909	
OR51E2	(Z)-Non-2-enal	-4.0	-3.95655	
OR51E2	2-Octenal	-4.3	-4.27804	
OR51E2	Bis(methylthio)methane	-2.4	-2.37581	
OR51E2	Dimethyl Disulfide	-2.1	-2.10506	
OR51E2	Dipropyl disulfide	-3.5	-3.51037	
OR51E2	Dimethyl trisulfide	-2.3	-2.34385	
OR51E2	3,6-nonadienal	-4.3	-4.2542	
OR51E2	Ethyl Hexanoate	-4.1	-4.05143	
OR51E2	Butyric acid	-3.6	-3.68966	Yes[7]
OR51E2	Cis-3-Hexen-1-ol	-4.1	-4.20676	
OR51E2	Hexyl acetate	-4.3	-4.27047	
OR51E2	Pentyl propionate	-4.2	-4.17755	
OR51E2	2-Methylbutyraldehyde	-3.6	-3.61929	
OR51E2	Isobutyric acid	-3.6	-3.71422	
OR51E2	Ethyl Valerate	-4.1	-4.05922	
OR51E2	Propionic acid	-3.3	-3.38438	Yes[7]
OR51G2	2-Methyl-1-Butanol	-3.7	-3.77613	
OR51G2	Isoamyl alcohol	-3.6	-3.70222	
OR51G2	2-Methylbutanoic acid	-4.2	-4.35227	
OR51G2	Trans-2-nonenal	-4.6	-4.55512	
OR51G2	(Z)-Non-2-enal	-4.6	-4.58044	
OR51G2	2-Octenal	-4.4	-4.45056	
OR51G2	Bis(methylthio)methane	-2.2	-2.20941	
OR51G2	Dimethyl Disulfide	-2.0	-1.99088	
OR51G2	Dipropyl disulfide	-3.6	-3.61633	
OR51G2	Dimethyl trisulfide	-2.2	-2.24889	
OR51G2	3,6-nonadienal	-4.8	-4.83111	
OR51G2	Ethyl Hexanoate	-4.5	-4.47563	
OR51G2	Butyric acid	-3.8	-3.90581	
OR51G2	Cis-3-Hexen-1-ol	-4.1	-4.16359	
OR51G2	Hexyl acetate	-4.4	-4.42466	
OR51G2	Pentyl propionate	-4.6	-4.63437	
OR51G2	2-Methylbutyraldehyde	-3.7	-3.67565	
OR51G2	Isobutyric acid	-3.9	-4.0064	
OR51G2	Ethyl Valerate	-4.4	-4.37418	
OR51G2	Propionic acid	-3.3	-3.40627	

for its reliability in simulating biological macromolecules. The water molecules were represented using the TIP3P model, a three-point water model known for its efficiency and accuracy in molecular dynamics simulations. This comprehensive setup ensures a realistic representation of the receptor's native environment, crucial for studying its behavior and interactions.

Protocol: Molecular Dynamic Simulations

An extended CHARMM-GUI-suggested workflow for equilibration and production runs of transmembrane proteins was initiated using GROMACS-2023.2[10] in a 5-step process. The protocol was divided into 7-steps namely, energy minimization, annealing, 3-steps of NVT equilibration, NPT equilibration, and the final *MD* run:

- **Step-1 (Energy Minimization):** 50,000 nsteps of steepest descent minimization, restraining the protein backbone (k = 4,000 kJ/mol/nm2) and sidechains (k = 2,000 kJ/mol/nm2) and dihedrals (k = 1,000 kJ/mol/rad2)

- **Step-2 (Simulated Annealing):** Performed to bring the system to the desired temperature and to avoid system explosion during equilibration. The temperature of the system was brought down to 300K in 1ns. Berendsen *pcoupl*, V-rescale *tcoupl*, Semi-isotropic *pcoupltype*.

- **Step-3,4 and 5 (NVT-1 equilibration):** Three NVT runs were performed to decrease restraints on the membrane slowly. The thermostat used was V-rescale for all three NVT steps. 125 ps of MD with a time step of 1 fs, restraining the protein backbone (k = 1,000 kJ/mol/nm2) and sidechains (k = 500 kJ/mol/nm2), lipid phosphate groups (k = 400 kJ/mol/nm2) and dihedrals (k = 200 kJ/mol/rad2).

- **Step-6 (NPT equilibration):** The Parrinello-Rahman barostat was used and 10ns

equilibration was performed at T = 310K. C-rescale *pcoupl*, V-rescale *tcoupl*, Semi-isotropic *pcoupltype*.

- **Step-7 (MDrun):** 100ns unbiased simulation using Nosè-Hoover thermostat coupled with the Parrinello Rahman barostat to maintain constant Temperature = 310K and Pressure = 1 bar.

Following the molecular dynamics (MD) simulations, the trajectory was preprocessed to ensure accurate and meaningful analyses. First, periodic boundary condition (PBC) artifacts were removed by applying `gmx trjconv` with the `-pbc mol` option, which ensures that entire molecules remain intact and are not split across the simulation box boundaries. The protein was then centered within the simulation box to maintain a coherent frame of reference. Subsequently, the trajectory was fitted using rotational and translational adjustments `(-fit rot+trans)` to eliminate global motions, allowing the focus to remain on the internal dynamics of the system. Preprocessing ensures that subsequent analyses accurately reflect the intrinsic behavior of the protein, free from distortions caused by pre-boundary conditions or extraneous movements.

References

(1) Mainland, J. D.; Li, Y. R.; Zhou, T.; Liu, W. L. L.; Matsunami, H. Human Olfactory Receptor Responses to Odorants. *Scientific Data* **2015**, *2*, 150002.

(2) Bushdid, C.; de March, C. A.; Fiorucci, S.; Matsunami, H.; Golebiowski, J. Agonists of G-Protein-Coupled Odorant Receptors Are Predicted from Chemical Features. *The Journal of Physical Chemistry Letters* **2018**, *9*, 2235–2240.

(3) Dunkel, A. e. a. Nature's Chemical Signatures in Human Olfaction: A Foodborne Perspective for Future Biotechnology. *WILEY-VCH Verlag GmbH Co. KGaA, Weinheim* **2014**, *53*, 28.

(4) Halperin Kuhns, V. L.; Sanchez, J.; Sarver, D. C.; Khalil, Z.; Rajkumar, P.; Marr, K. A.; Pluznick, J. L. Characterizing Novel Olfactory Receptors Expressed in the Murine Renal Cortex. *American Journal of Physiology-Renal Physiology* **2019**, *317*, F172–F186.

(5) Li, S.; Ahmed, L.; Zhang, R.; Pan, Y.; Matsunami, H.; Burger, J. L.; Block, E.; Batista, V. S.; Zhuang, H. Smelling Sulfur: Copper and Silver Regulate the Response of Human Odorant Receptor OR2T11 to Low-Molecular-Weight Thiols. *Journal of the American Chemical Society* **2016**, *138*, 13281–13288.

(6) Pronin, A.; Slepak, V. Ectopically Expressed Olfactory Receptors OR51E1 and OR51E2 Suppress Proliferation and Promote Cell Death in a Prostate Cancer Cell Line. *Journal of Biological Chemistry* **2021**, *296*.

(7) Billesbølle, C. B.; March, C. A. d.; van der Velden, W. J. C. e. Structural Basis of Odorant Recognition by a Human Odorant Receptor. *Nature* **2023**, *615*.

(8) Jo, S.; Kim, T.; Iyer, V. G.; Im, W. CHARMM-GUI: A web-based graphical user interface for CHARMM. *Journal of Computational Chemistry* **2008**, *29*, 1859–1865.

(9) Brooks, B. R. et al. CHARMM: The biomolecular simulation program. *Journal of Computational Chemistry* **2009**, *30*, 1545–1614.

(10) Abraham, M. J.; Murtola, T.; Schulz, R.; Páll, S.; Smith, J. C.; Hess, B.; Lindahl, E. GROMACS: High performance molecular simulations through multi-level parallelism from laptops to supercomputers. *SoftwareX* **2015**, *1-2*, 19–25.
